# Investigating Sensorimotor Decision-Making in Depressed Individuals Using a Bayesian Approach

**DOI:** 10.5334/cpsy.195

**Published:** 2026-10-09

**Authors:** Xin Song, Mathi Manavalan, Julia Griem, Tobias Nolte, Peter Fonagy, Iris Vilares

**Affiliations:** 1 Department of Psychology, University of Minnesota, 75 E River Road, Minneapolis, MN 55455, United States of America; 2 Research Department of Clinical, Educational and Health Psychology, University College London, London, United Kingdom; 3 Department of Forensic and Neurodevelopmental Sciences, Institute of Psychiatry, Psychology and Neuroscience, King’s College London, United Kingdom; 4 Anna Freud, London, United Kingdom

**Keywords:** Bayesian Decision-Making, Depression, Prior, Likelihood

## Abstract

Depression is among the most prevalent and disabling mental health conditions worldwide. It is associated with low mood and motivation, cognitive inefficiencies, altered reward and punishment learning, and broader executive dysfunction that can disrupt daily functioning. Depression has also been linked to differences in sensory perception, sensitivity, and processing speed. However, how depression affects sensorimotor decision-making under uncertainty remains comparatively understudied. In this study, we examined how individuals with depression process and weigh prior information relative to current sensory evidence during sensorimotor decisions. Participants completed a visuomotor coin-catching task, and behavior was analyzed within a Bayesian Decision Theory framework. We tested whether reliance on current information differed between depressed and non-depressed individuals, and whether any differences depended on uncertainty in the prior or the sensory signal. Across behavioral and computational analyses, both groups combined prior knowledge and sensory input in a qualitatively Bayesian manner, placing greater weight on current information when it was less uncertain and/or when prior information was more uncertain. The groups did not differ significantly in overall relative reliance on current information. Despite these broad similarities, more subtle differences were apparent. Model-based analyses indicated that Bayesian models outperformed heuristic alternatives in both groups, but model fit was poorer in the depressed group. Both groups adjusted sensory weighting as prior uncertainty changed, but non-depressed participants showed about twice the shift observed in depressed individuals, although this difference was not statistically significant. Together, these findings suggest that depression does not eliminate Bayesian-like integration of priors and sensory evidence, but may potentially reduce sensitivity to changes in prior reliability. Future work should test whether individual differences in symptom profiles, task demands, and cognitive flexibility moderate these effects.

## Introduction

Depression is among the leading causes of disability worldwide, with estimates suggesting that more than 250 million people are affected globally (GBD 2019 Mental Disorders Collaborators, 2022). Heterogeneous in nature (Bahrami et al., 2021; Fang et al., 2020), depression frequently remains underdiagnosed (Williams et al., 2017), contributes to substantial subjective distress (Angst et al., 2010; Bathina et al., 2021), socioeconomic burden (Greenberg et al., 2015), and, despite the availability of effective treatments, is characterized by high relapse rates (Zhang et al., 2018) and treatment resistance in a substantial subgroup of patients (Jaffe et al., 2019). Depression manifests across multiple domains of everyday functioning, including heightened negative affect (Geisser et al., 2000; Wichers et al., 2007), diminished positive affect (Admon & Pizzagalli, 2015; Cooper et al., 2018; Rizvi et al., 2016; Treadway & Zald, 2013), and reduced reward motivation and anticipation (Admon & Pizzagalli, 2015; Eshel & Roiser, 2010; Grahek et al., 2019; Höflich et al., 2018; Paulus & Yu, 2012; Pizzagalli, 2014; Rizvi et al., 2016; Roiser & Sahakian, 2013). Converging evidence links depression to disturbances in reward processing that shape value-based decision-making strategies. However, much of this work relies on affect-laden stimuli and reward-driven paradigms, leaving open the question of whether depression alters more fundamental aspects of decision-making — namely, individuals’ sensory perception and its integration with prior knowledge.

Indeed, alongside alterations in reward learning and processing, individuals with depression often report changes in sensory perception. Prior work indicates that depression can be associated with atypical visual perception, reduced registration of sensory input, heightened sensory sensitivity, and increased sensory-avoidance behaviors (Fitzgerald, 2013; Liss et al., 2005; Paquet et al., 2022; Serafini et al., 2017; Wang et al., 2020). Depression has also been linked to slower visual processing and encoding, as well as prolonged evidence accumulation, even when sensory decision-making is broadly preserved (Lawlor et al., 2020; Zaremba et al., 2019; Zarrinpar et al., 2006). Systematic reviews support these observations. Reviews of 252 and 37 studies, respectively, have reported persistent cognitive dysfunction and slower processing speed even after remission from a depressive episode, as well as impaired task performance on measures such as the Trail Making Test and Stroop Test, consistent with delayed information processing in depression (Nuño et al., 2021; Semkovska et al., 2019).

Collectively, these findings suggest that depression is not solely an affective disorder; altered sensory perception and associated sensorimotor decision-making may be a measurable feature of depression that warrants further investigations (Canbeyli, 2022; Desseilles et al., 2009, 2011; Serafini et al., 2017). Notably, altered sensory sensitivity and sensory gating have been reported to precede depression and may operate as risk markers (Hermesdorf et al., 2016; Klauenberg et al., 2008; Storozheva et al., 2021). Yet research on lower-level perceptual decision-making in depression remains limited. Two studies reported that individuals with depression had difficulty detecting non-emotional stimuli presented at a fixed central visual location, potentially reflecting abnormal filtering of irrelevant peripheral input (Desseilles et al., 2009, 2011). More recently, attention has turned to the interplay between top-down and bottom-up processing of lower-level, non-emotional stimuli. In related work, individuals with neurological disorders such as Parkinson’s disease and individuals with psychosis-related symptoms have shown altered reliance on bottom-up sensory information versus top-down prior information in simple visuomotor tasks, relative to neurotypical controls (Goodwin et al., 2023; Vilares & Kording, 2017). Whether depression similarly alters the balance between top-down and bottom-up processing in non-emotional sensorimotor decision-making remains unclear.

To address these gaps, we examined top-down and bottom-up competition in sensorimotor decision-making in depression through the lens of a Bayesian framework. Bayesian Decision Theory proposes that decisions reflect the integration of prior knowledge (historical information and beliefs) with current sensory input, or likelihood information. Decisions are relatively more top-down when priors exert greater influence and relatively more bottom-up when likelihood information predominates. Critically, the normative Bayesian account predicts that priors and likelihoods should be weighted according to their relative uncertainty. This approach has been influential in research on sensorimotor control, where healthy individuals often appear to combine uncertain prior information and sensory evidence in a manner consistent with Bayesian integration, generating posterior estimates that are closely predicted by Bayesian statistics (Körding & Wolpert, 2006; Vilares & Kording, 2017). To our knowledge, it remains unclear whether individuals with depression employ a non-optimal or otherwise altered strategy when integrating prior and likelihood information during non-social, non-affectively charged sensorimotor decision-making. In addition, we sought to test whether purported alterations in sensory gating and processing speed extend to lower-level sensorimotor decision-making. To this end, we employed a visuomotor task to evaluate how individuals with depression weigh and use prior and sensory information relative to non-depressed individuals.

We hypothesized that individuals with depression would depend less on visuospatial sensory information, consistent with slower processing speed and protracted evidence accumulation. We expected this to manifest as greater reliance on prior information, reduced weighting of sensory evidence, and attenuated sensitivity to changes in prior and sensory uncertainty.

## Methods

### Participants

Participants for this pilot study were recruited through two streams. The first stream comprised 21 individuals (7 males, 14 females; mean age = 27.43 ± 7.00 years) with depression and/or anxiety who were on the waiting list for high-intensity CBT within the NHS Talking Therapies service (formerly IAPT). The second stream comprised 20 individuals (10 males, 9 females, 1 transgender; mean age = 23.78 ± 3.94 years) with no current mental health or personality disorder diagnosis and not undergoing psychological treatment, who volunteered after seeing online advertisements. All participants were recruited as part of a larger study examining decision-making in psychopathology; data from 18 individuals without a diagnosis have been reported previously (Manavalan et al., 2024).

Participants with a current or past diagnosis of a psychotic disorder or recent psychotic episode, addiction or substance use disorder, neurological disorder, or history of traumatic brain injury were excluded. Both groups completed the Depressive Experiences Questionnaire (Blatt et al., 1995) and Beck’s Depression Inventory-II (Beck et al., 1996) at baseline. Participants were classified as depressed or non-depressed based on BDI-II total score, with scores >13 indicating depression and scores ≤13 indicating no depression. This cutoff followed standard BDI-II severity bands (0–13 minimal, 14–19 mild, 20–28 moderate, 29–63 severe). We verified robustness of the split with a different threshold, which resulted in an identical group composition (see SI for details). Questionnaire data were unavailable for two referred participants (stream 1), who were excluded. Of the referred participants with questionnaire data (n = 19), all met the depressed criterion. Among controls, five had BDI-II scores >13 and were classified as depressed, and 15 had scores ≤13 and were classified as non-depressed. The final sample comprised 24 individuals in the depressed group (7 males, 16 females, 1 transgender; mean age = 28.8 ± 8.27 years) and 15 individuals in the non-depressed group (8 males, 7 females; mean age = 23.3 ± 4.17 years). Participants received £10/hour compensation and provided written informed consent prior to participation. The study was approved by the London–Queen Square Research Ethics Committee (16/LO/0077).

### Task and stimuli

We employed a visuomotor coin-catching task (Vilares et al., 2012) to assess decision-making under uncertainty while independently manipulating uncertainty in prior information and sensory evidence. Participants were instructed to imagine a person tossing a coin into the center of a pond, producing five “water splashes” as the coin submerged (Figure 1; light-blue dot cloud), with additional dark-gray background dots (Figure 1). The coin’s exact location was not initially visible. Participants estimated its position by moving a “net” (Figure 1, blue bar) to the location where they believed the hidden coin was located; the net’s starting position was randomized on each trial. After participants positioned the net and pressed Enter to submit their response, the coin’s true location was revealed. Participants earned one point when their final net position covered at least half of the coin. No additional reward feedback was provided for correct responses, and incorrect responses were not penalized (see Figure 1 for the trial procedure).

**Figure 1:**
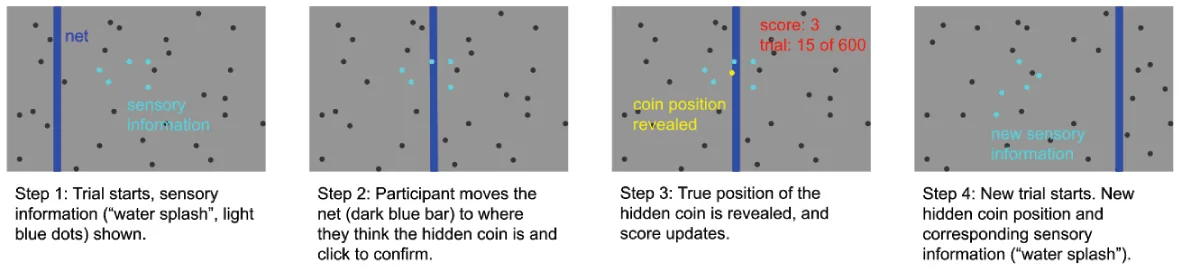
Experimental procedure of the coin-catching task. Left: Test screen and procedure: a thrower throws a coin into a pond, aiming at the center. When the coin falls into the pond, it makes 5 splashes (blue dots on the screen, which is our sensory information). Middle: Participants are told to move the blue net to guess the position of the hidden coin. Right: Once confirmed, the true coin location (yellow dot) is then revealed.

The task comprised four blocks. The standard version included 150 trials per block (600 total trials). A shortened version included 120 trials per block (480 total trials) to accommodate restlessness and was implemented because no behavioral differences were observed between participants completing 120 versus 150 trials per block. Due to a technical implementation error, 17 depressed participants and 1 non-depressed participant completed the 600-trial version, whereas 7 depressed participants and 14 non-depressed participants completed the 480-trial version. Post hoc sensitivity analyses indicated no significant differences between task versions regarding the main measure of this task (sensory weights), and data were therefore combined. We also included task version as a covariate for the main analyses in the paper; see Results for details.

The task used a 2 × 2 design with two levels of prior uncertainty and two levels of likelihood (sensory) uncertainty, yielding four conditions (Figure 2). To define the prior, participants were told that the “thrower” always aimed for the pond’s center (prior mean μ_P = 0.5 in screen coordinates). Prior variance was either large (*σ_P_*^2^ = *0.075^2^*) or small (*σ_P_*^2^ = *0.025^2^*); participants were told that the thrower could be “good” (smaller variance) or “bad” (larger variance) at aiming. Likelihood uncertainty was manipulated by varying the spread of the splash locations (Figure 2). On each trial, each splash was sampled from a Gaussian distribution centered on the coin’s true position, with either small variance (*σ_l_*^2^ = *0.025^2^* * 5) or large variance (*σ_L_*^2^ = *0.025^2^* * *5*). Wider dot-cloud spread indicated higher likelihood variance (less reliable sensory information), whereas narrower spread indicated lower likelihood variance (more reliable sensory information). Only horizontal (x-axis) dispersion was used in analyses; dispersion was counterbalanced across axes such that a larger spread on the x-axis corresponded to a smaller spread on the y-axis.

**Figure 2:**
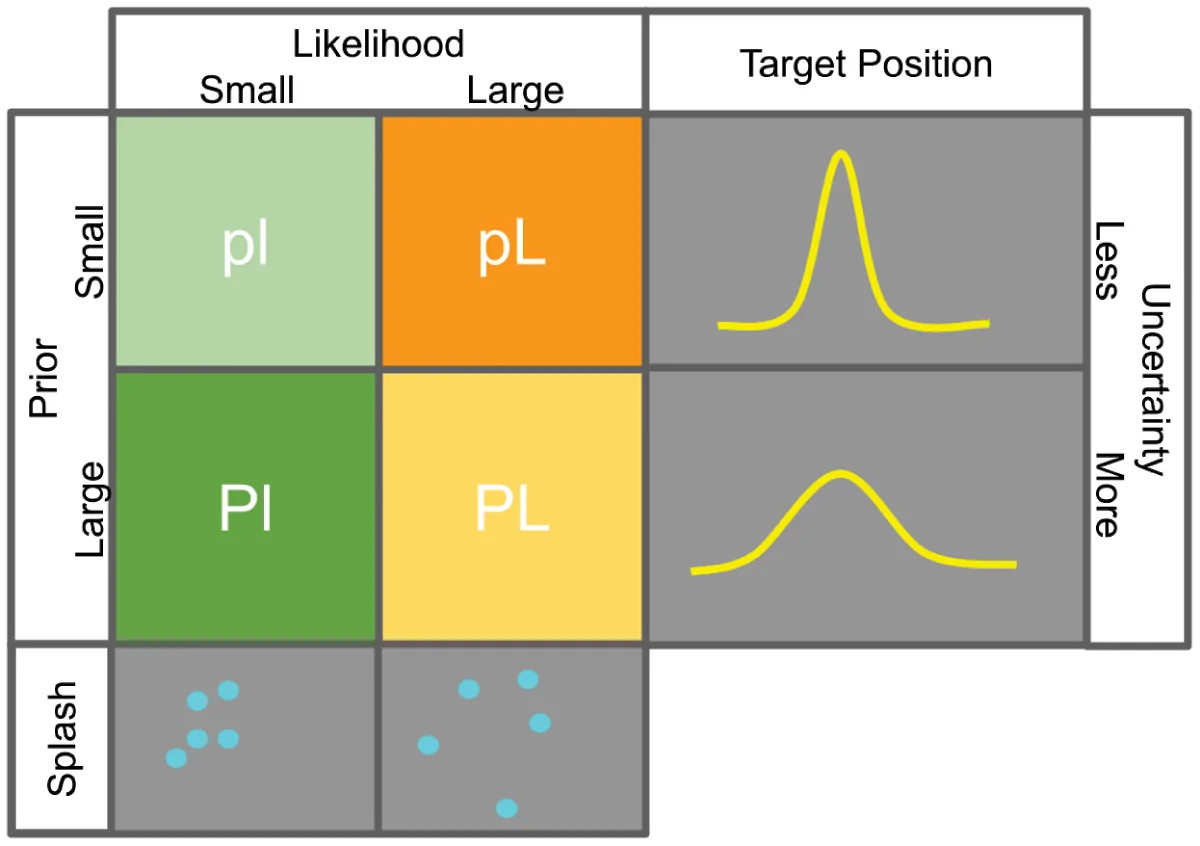
The four conditions of the experiment (figure adapted from Vilares et al. (2012). The experiment has a two-by-two factorial design with two types of prior (p = small prior uncertainty; P = large prior uncertainty) and two types of likelihood (l = small likelihood uncertainty; L = large likelihood uncertainty). The larger conditions are the ones with more uncertainty.

The thrower identity remained constant within each block and changed only between blocks. Thus, prior variance remained constant within blocks and was learned across trials, whereas likelihood variance was directly observable on each trial from the dot-cloud spread. Small and large prior variances alternated across blocks (two blocks per prior condition). Likelihood variance was randomized trial-by-trial with equal numbers of trials per condition. This task dissociates effects of prior and likelihood uncertainty and has shown good reliability (Goodwin et al., 2023; Manavalan et al., 2024).

### Modeling Posterior Coin Estimation

The Bayesian Optimal Observer model provides a normative account of how prior knowledge and sensory evidence can be combined to form a posterior estimate of a sensorimotor target (Körding & Wolpert, 2006). The posterior mean is given by:


$$X_{\text{est}}\;\text{=}\;\sigma_{L}^{2}\text{/}(\sigma_{L}^{2}+\sigma_{P}^{2})\mu_{P}+\sigma_{P}^{2}\;\text{/}\;(\sigma_{L}^{2}+\sigma_{P}^{2})\mu_{L}$$

In the current coin-catching task, *X_est_* represents participants’ estimated coin location of each trial, *σ_L_^2^* and *μ_L_* are the variance and mean of the likelihood/sensory information (the spread and centroid of the dot-cloud in each trial), and *σ_L_^2^* and *μ_L_* are the variance and mean of the prior (previous distribution of coin location and the center of the screen). Of note, *σ_P_^2^*/(*σ_L_^2^* + *σ_P2_*) presents the Bayesian-optimal weight on current sensory information. Under Bayes-optimal behavior, the empirical sensory weight, estimated by regressing participants’ location estimates on the dot-cloud centroid across trials, should match the Bayesian-optimal weight. We therefore used sensory weight as a quantitative index of reliance on sensory evidence.

To further characterize the strategies participants used to estimate coin location, we fit a set of Bayesian and non-Bayesian models to trial-wise responses and evaluated model fit using MSE and adjusted (R^2). Detailed model specifications and Bayesian-optimal sensory weights are provided in the Supplemental Information, and model results are reported in Table 1.

**Table 1:** Model performance. Mean square error (MSE), and adjusted R-squared (*R*^2^) for each model. The results were calculated using the full dataset including all trials across all participants. Negative *R*^2^ indicates that the model performs worse than a horizontal line (i.e., worse than simply predicting the mean of the data).


MODEL	MSE	*R^2^*

M1. Sense Only	0.003728796	0.2396081

M2. Prior Mean Only	0.004908467	–0.0009554653

M3. Previous Coin Location	0.007733575	–0.5770636

M4. Bayesian Optimal	0.002933068	0.4018763

M5. Bayes Optimal Prior Learned	0.002901039	0.4084076


## Results

### Sensory reliance and Bayesian decision-making

To test whether depression affected the relative reliance on sensory versus prior information, we quantified how strongly participants’ coin-location estimates were driven by the dot-cloud centroid on each trial (i.e., the current sensory likelihood). Under Bayesian Decision Theory, reliance on one source of information should increase as the other becomes more uncertain. We operationalized reliance on sensory information by regressing each participant’s estimated coin location on the dot-cloud centroid across trials; the slope of this regression was termed the sensory weight (Vilares et al., 2012). A sensory weight of 1 indicates exclusive reliance on current sensory information, whereas a sensory weight of 0 indicates no reliance on sensory information (and, under the assumption that participants relied solely on priors and/or likelihoods, implies exclusive reliance on prior information).

In both groups, mean sensory weight differed significantly from 0 (depressed: *M* = 0.61, *SD* = 0.20, *t*(23) = 14.60, *p* < 10^–12^); non-depressed: *M* = 0.58, *SD* = 0.17, *t*(14) = 13.56, *p* < 10^–8^; one-sample t-tests), indicating substantive use of sensory information. Sensory weight also differed significantly from 1 in both groups (depressed: *t*(23) = –9.45, *p* < 10^–8^; non-depressed: *t*(14) = –9.77, *p* < 10^–6^), indicating that decisions were not based solely on sensory evidence and were consistent with integration of sensory and prior information. Contrary to our hypothesis, there was no difference in overall sensory weights between groups (*t*(37) = 0.43, *p* = 0.67; Cohen’s *d* = 0.14; Figure 3). Sensitivity analyses controlling for task version confirmed that sensory weight remained significantly different from both 0 and 1 in each group (all *p*s < .001), and the non-significant group difference was unchanged (*F*(1, 36) = 0.14, *p* = .71, *d* = 0.16).

**Figure 3:**
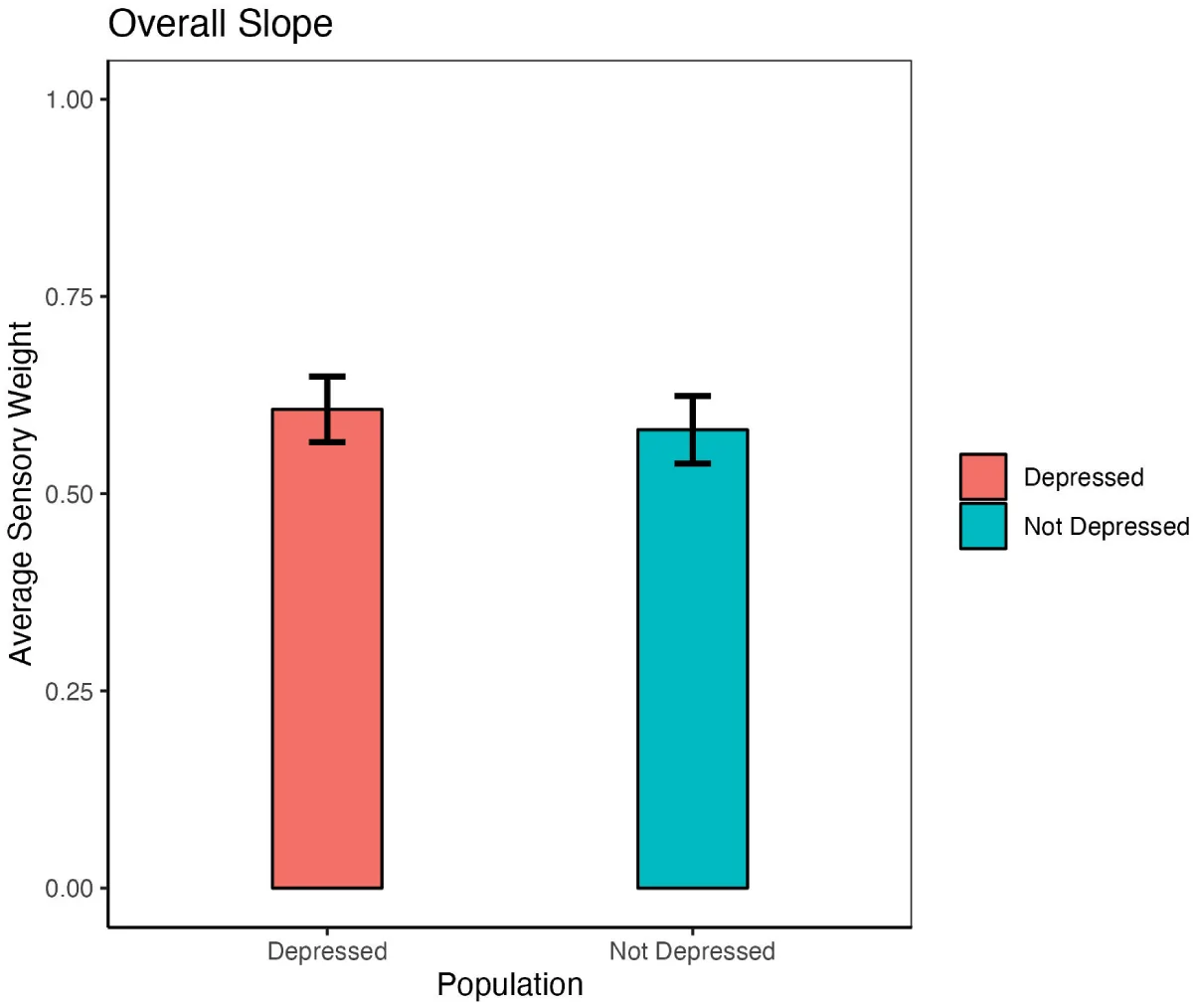
Average sensory weight for the two experimental groups. Both the depressed and non-depressed groups exhibit sensory weights significantly different from 1 (relying solely on sensory information) and from 0 (relying solely on prior information). There were no significant differences between the groups.

We then examined whether sensory reliance varied across experimental conditions using a linear mixed-effects model with prior uncertainty, likelihood uncertainty, and group as fixed effects and participant as a random effect. Our initial model included by-participant random intercepts only, which yielded a significant prior uncertainty × group interaction (*F*_1,111_ = 7.00, *p* = 0.009). Because both uncertainty factors were manipulated within participants, we refit the model including by-participant random slopes for prior and likelihood uncertainty; this specification provided a substantially better fit (χ²(5) = 25.45, *p* < 0.001), and we report it throughout. Fixed-effect estimates were identical across the two models, which differ only in the precision with which those effects are estimated. There were significant main effects of prior uncertainty (*F*_1,37_ = 38.72, *p* < 10^–^^6^) and likelihood uncertainty (*F*_1,37_ = 131.25, *p* < 10^–13^). Participants in both groups showed lower sensory weights when prior uncertainty was small, consistent with greater reliance on priors when they were more reliable (see Figure 4). Participants also showed higher sensory weights when likelihood uncertainty was small, consistent with greater reliance on sensory information when that was more reliable (see Figure 4). The prior × likelihood interaction was significant (*F*_1,37_ = 4.72, *p* = 0.036), reflecting a larger effect of prior uncertainty when sensory evidence was reliable (*Δ* = 0.155, 95% CI [0.107, 0.202]) than when it was unreliable (*Δ* = 0.107, 95% CI [0.060, 0.155]), and there was no main effect of group (*F*_1,37_ = 0.22, *p* = 0.64). The prior uncertainty × group and likelihood uncertainty × group interactions were marginally significant/non-significant (*F*_1,37_ = 4.07, *p* = 0.051; *F*_1,37_ = 3.26, *p* = 0.079, respectively). The three-way interaction was not significant (*F*_1,37_ = 0.18, *p* = 0.68). To decompose the prior × group interaction, we estimated the simple effect of prior uncertainty within each group using estimated marginal means averaged across likelihood conditions. Both groups showed a significant increase in sensory weighting when prior uncertainty was large (i.e., when priors were less reliable): the non-depressed group adjusted by *Δ* = 0.173 (95% CI [0.106, 0.240], *t*(37) = 5.25, *p* < 0.001), while the depressed group adjusted by *Δ* = 0.088 (95% CI [0.036, 0.141], *t*(37) = 3.39, *p* = 0.002). The difference between these two effects corresponded to approximately twice the adjustment in the non-depressed group, but was only marginally significant (*Δ* = 0.085, 95% CI [–0.0004, 0.170], *t*(37) = 2.02, *p* = 0.051). This estimate is consistent with the participant-level sensitivity analysis reported in the Supplemental Information (*t*(37) = –2.0, *p* = 0.051), which tests a related contrast but without taking into account random participant effects (see Supplemental Information and Fig. S1). As described in the Methods, depressed participants were recruited from both a referred (NHS) and a community stream; we conducted two sensitivity analyses to assess whether recruitment stream affected these results. When recruitment stream was included as a covariate, all effects were essentially unchanged (prior × group: *F*_1,37_ = 4.07, *p* = 0.051; main effects of prior and likelihood both *p* < 10^–6^), and recruitment stream itself was not significant (*F*_1,36_ = 2.23, *p* = 0.14). When the five community-recruited depressed participants were excluded entirely, the estimated group difference was similar in magnitude and direction (prior vs group interaction term: *β* = –0.084, compared with *β* = –0.094 in the full sample) but was no longer statistically distinguishable from zero (*F*_1,36_ = 2.33, *p* = 0.14)), consistent with the reduced power of the smaller depression sample.

**Figure 4:**
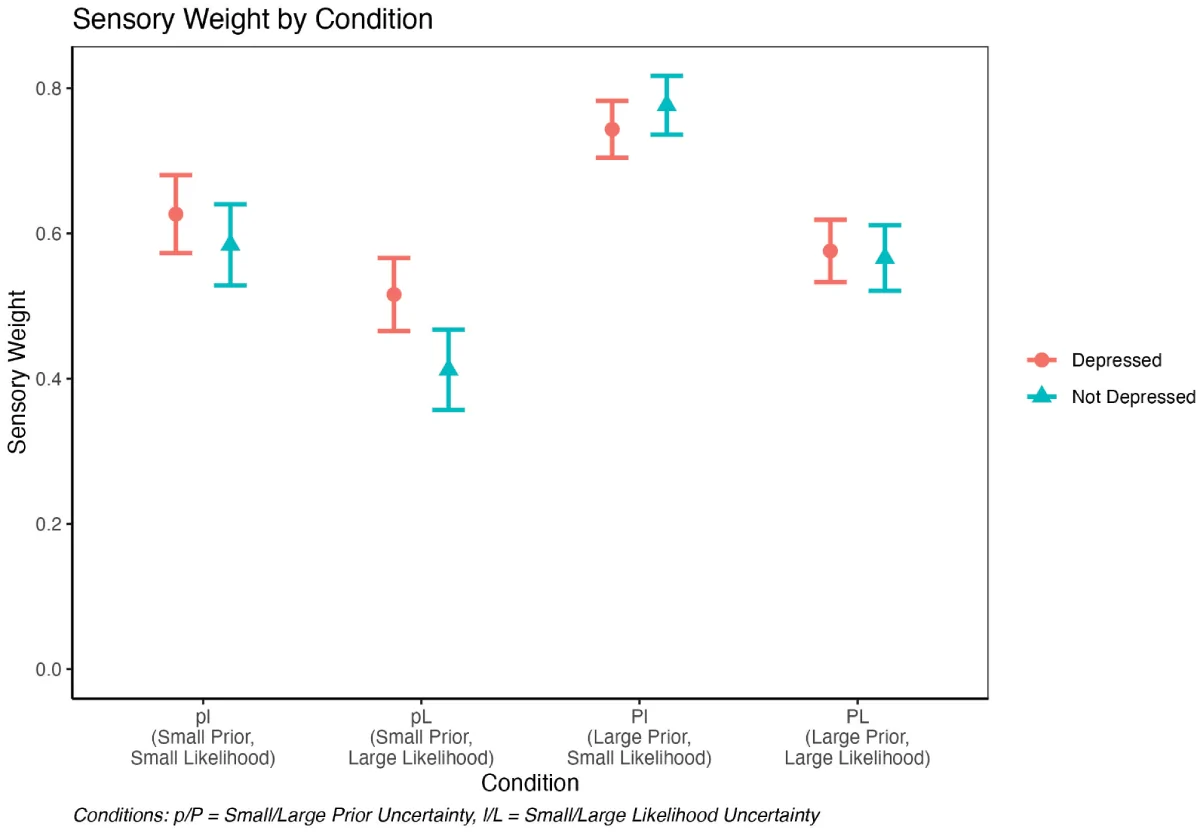
Mean sensory weights ± s.e.m. for depressed and non-depressed participants by condition. Conditions (x-axis), left to right: small prior/small likelihood uncertainty (*pl*), small prior/large likelihood uncertainty (*pL*), large prior/small likelihood uncertainty (*Pl*), and large prior/large likelihood uncertainty (*PL*). The y-axis shows mean sensory weight per condition, plotted separately by group. A mixed-effects model with prior, likelihood, and group as fixed effects and participant as a random effect showed significant main effects of prior and likelihood uncertainty, but no main effect of group. In both groups, sensory weight was lower when the prior was more reliable and when the likelihood was less reliable. Although there was no main effect of group, there was a significant prior × group interaction: the non-depressed group showed a larger reduction in sensory weighting under low prior uncertainty.

Including age and gender as covariates did not alter this interaction, and neither covariate reached significance. We also included task version as a covariate (480 vs. 600 trials) to account for potential training effects due to differential exposure but found no main effect of version (*F*_1,36_ = 0.17, *p* = 0.69) and no interactions between version and the experimental factors (all *ps* > 0.05). This pattern indicates that the observed sensory-weight effects were robust across task versions. Analyses based on condition-level averages (i.e., with only one value per participant, not taking into account participant-specific random effects) showed a similar pattern (see Supplemental Information and Figure S1). Overall, both groups behaved in a qualitatively Bayesian manner, placing greater weight on the less uncertain information source, and the non-depressed group showed a marginally larger adjustment in sensory weighting as prior uncertainty changed, though this group difference was only marginally significant (Figure 4). See Supplemental Information for comparison between participants’ sensory weights and Bayesian-optimal sensory weights, analyses of changes in sensory weights over time (Figure S2), and reaction to trial-by-trial changes in likelihood uncertainty (Figure S3).

### Performance and Reaction Times

To test whether depressed participants differed from non-depressed participants in task performance, we analyzed accuracy across the four experimental conditions. Performance was defined as the proportion of correct trials within each condition. We fit a linear mixed-effects model with prior uncertainty (large vs. small), likelihood uncertainty (large vs. small), and group (non-depressed vs. depressed) as fixed effects, and participant as a random effect. There were significant main effects of prior uncertainty (*F*_1,111_ = 78.95, *p* < 0.01) and likelihood uncertainty (*F*_1,111_ = 278.04, p < 0.01), indicating higher accuracy when either prior or likelihood uncertainty was small. There was no main effect of group (*F*_1,37_ = 1.36, *p* = 0.25), and no two-way or three-way interactions (all *p*s > 0.35; Figure 5, left). We found a main effect of task version when task version was included as a covariate *F*_1,36_ = 5.45, *p* = 0.025), such that participants who completed the 600-trial version performed better overall, likely reflecting additional practice. Notably, after controlling for task version, the main effect of group became significant (*F*_1,36_ = 5.87, *p* = 0.021), with non-depressed participants outperforming depressed participants. Because depressed participants were disproportionately assigned to the longer task version, the additional practice they received appears to have inflated their accuracy, masking an underlying group difference. Once this practice advantage was accounted for, a performance difference between groups emerged. All other effects remained consistent with the primary analysis (all interaction *p*s > 0.35).

**Figure 5:**
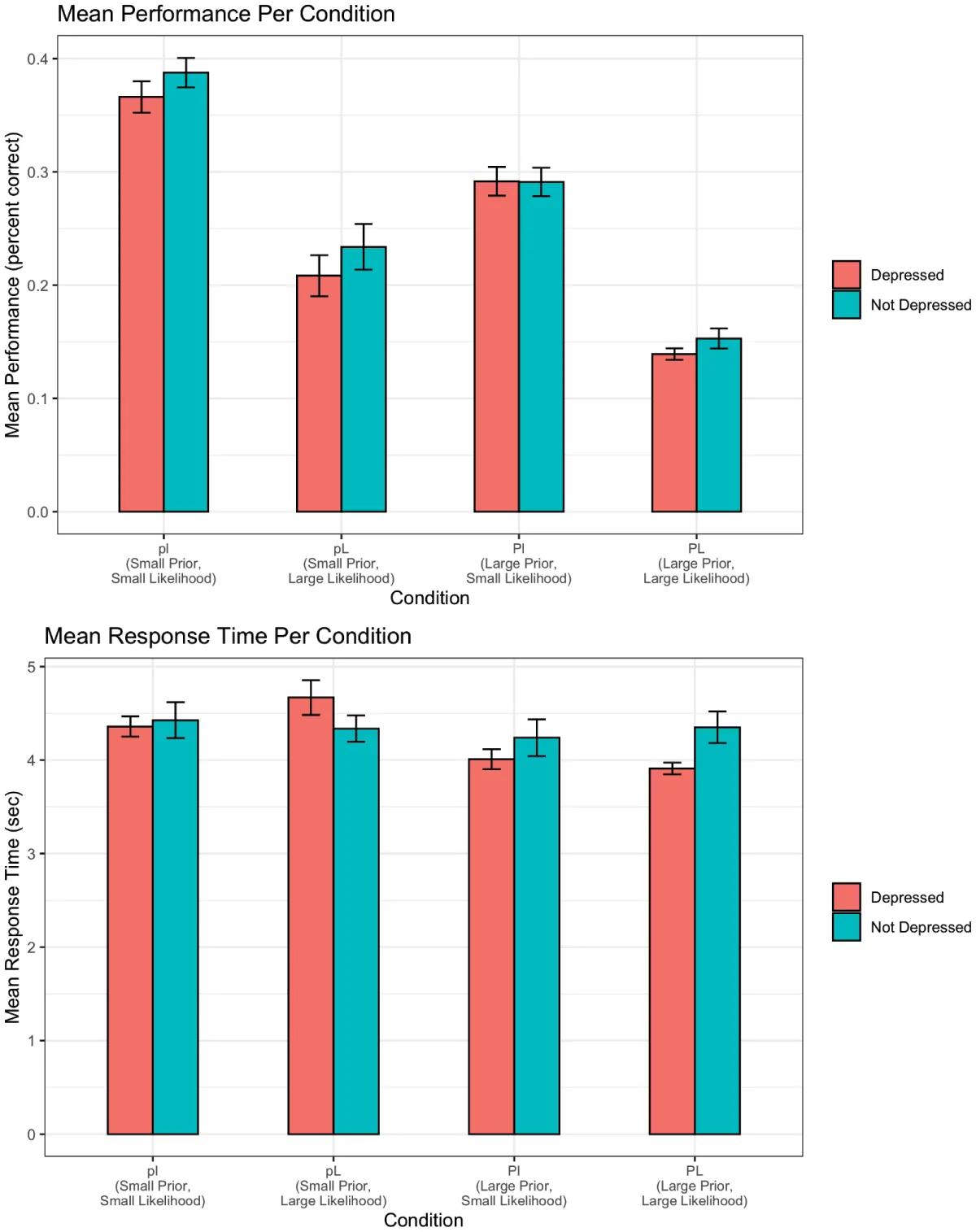
Task performance (percent correct; top panel) and response time (seconds; bottom panel) across conditions for the depressed (n = 24) and non-depressed (n = 15) groups. Performance was quantified as the proportion of correct trials per condition. Accuracy was highest in the pl condition relative to the other three conditions. No performance differences were detected between groups in the primary analysis; however, after controlling for task version, non-depressed participants showed significantly higher accuracy than depressed participants (see Results). Response time was averaged across trials within each condition. Both groups showed shorter response times under large prior uncertainty (conditions Pl and PL). Conditions are small prior and likelihood uncertainty (pl); small prior uncertainty, large likelihood uncertainty (pL), large prior uncertainty, small likelihood uncertainty (Pl), and large prior and likelihood uncertainty (PL).

We also examined response time, defined as the interval from trial onset to response submission, as an index of processing speed. Although processing speed and response time are conceptually distinct, response time is commonly used as a proxy for processing speed in speeded tasks (Buckhalt, 1991; Kyllonen & Zu, 2016). Response times were log-transformed prior to analysis to reduce positive skew. A linear mixed-effects model was used to test the effects of group, prior uncertainty, and likelihood uncertainty on response time across trials, with participant as a random effect. The only significant main effect was prior uncertainty (*F*_1,20835_ = 40.69, *p* < 0.001), such that participants responded faster when prior uncertainty was large, contrary to our prediction. There was a marginal group × prior uncertainty interaction (*F*_1,20835_ = 3.36, *p* = 0.067), suggesting a somewhat larger prior effect on response times in the depressed group. No other main effects or interactions reached significance. Results were unchanged when additionally controlling for age, gender, BDI-II severity or total score, and task version (480 trials vs 600 trials) (all *p*s > .05, see Supplemental Information).

### Computational Modeling

To characterize the strategies participants used to estimate coin location, we fit a set of non-Bayesian and Bayesian models to trial-wise responses (see Supplemental Information for model details). Models were used to predict each participant’s coin-location estimate on each trial. Because none of the models included free parameters, model accuracy was evaluated using MSE and adjusted *R*^2^. We prioritized parameter-free models to avoid overfitting, given our modest sample size; however, this means the models cannot capture individual differences in how participants combine information, which we address below. Model performance is summarized in Table 1.

Of the five models, three were heuristic accounts that did not combine prior and sensory information. The Sense Only model (M1) assumed estimates were determined solely by sensory evidence (i.e., the dot-cloud centroid). The Prior Mean Only model (M2) assumed responses were fixed at the prior mean (screen center). The Previous Coin Location model (M3) assumed responses depended only on the previous trial’s coin location. These heuristics were not intended as realistic accounts of behavior but rather as benchmarks representing reliance on a single information source. Their role was to test whether participants combined multiple cues rather than depending on any one source alone. Two Bayesian models assumed integration of prior and sensory information. The Bayes Optimal model (M4) computed the posterior estimate using the experimentally imposed prior and likelihood variances. The Bayes Optimal Prior Learned model (M5) extended M4 by allowing the prior variance to be learned and updated trial-by-trial (see Supplemental Information).

Across models, M5 achieved the lowest MSE and highest adjusted *R*^2^, indicating that it best captured participants’ trial-wise estimates (Table 1). M4 showed closely comparable performance to M5, and both Bayesian models outperformed the heuristic models (M1–M3). The heuristic models exhibited higher MSEs and lower adjusted *R*^2^, indicating a poorer account of participants’ estimation behavior.

A Kruskal–Wallis test indicated significant differences in MSE across models (*H*(4) = 83.059, *p* < 10^–15^). Post hoc pairwise Wilcoxon rank-sum tests with Benjamini–Hochberg correction showed that the Previous Coin Location model (M3) underperformed relative to all other models (all adjusted *p*s < 10^–15^), suggesting minimal influence of the immediately preceding coin location on current estimates. Both Bayesian models (M4 and M5) performed similarly (adjusted *p* > 0.05) and outperformed Prior Mean Only (M2), Previous Coin Location (M3), and Sense Only (M1) (adjusted *p* < 10^–15^ for M2; adjusted *p* < 0.001 for M1). Sense Only (M1) performed better than Prior Mean Only (M2) and Previous Coin Location (M3) (adjusted *p* < 0.05), but worse than the Bayesian models (adjusted *p* < 0.01). Collectively, these findings indicate that models combining prior and sensory information with uncertainty weighting best captured participants’ estimation behavior. That said, because the heuristic models each rely on only a single information source, their poor performance shows that participants combined multiple information sources in a way that is consistent with Bayesian integration, but it does not rule out other combination strategies that also use both sources (e.g., a fixed mixture that does not adjust to uncertainty); in other words, it is possible that any model that assigns non-zero weight to both prior and likelihood information would outperform M1-M3 on these measures, so the advantage of M4 and M5 may reflect the fact that they combine multiple information sources rather than the specific way in which they do so (e.g., a model that combines both prior and likelihood information and holds the relative weights constant across blocks and trials, regardless of their trial-wise uncertainty, might fit the data comparably well too). Future work with larger samples should test Bayesian models against such alternatives to determine whether the advantage of M4 and M5 reflects sensitivity to changes in uncertainty specifically, rather than information combination more generally.

To test whether model fit differed by group, we compared MSEs between depressed and non-depressed participants for each model using Wilcoxon rank-sum tests with Benjamini–Hochberg correction (Figure 6). Bayesian models fit the non-depressed group significantly better than the depressed group: both M5 and M4 yielded lower MSEs for non-depressed participants (adjusted *p* < 0.05 for each). The Sense Only model (M1) also fit the non-depressed group better than the depressed group (adjusted *p* < 0.05). M2 and M3 showed numerically better fits in the non-depressed group, but differences were not significant (*p*s > 0.1). Overall, model performance was systematically poorer in the depressed group, suggesting that depressed participants’ estimation behavior diverged further from both Bayes-optimal accounts and the simple heuristic alternatives considered here, consistent with the group differences in model fit illustrated in Figure 6.

**Figure 6:**
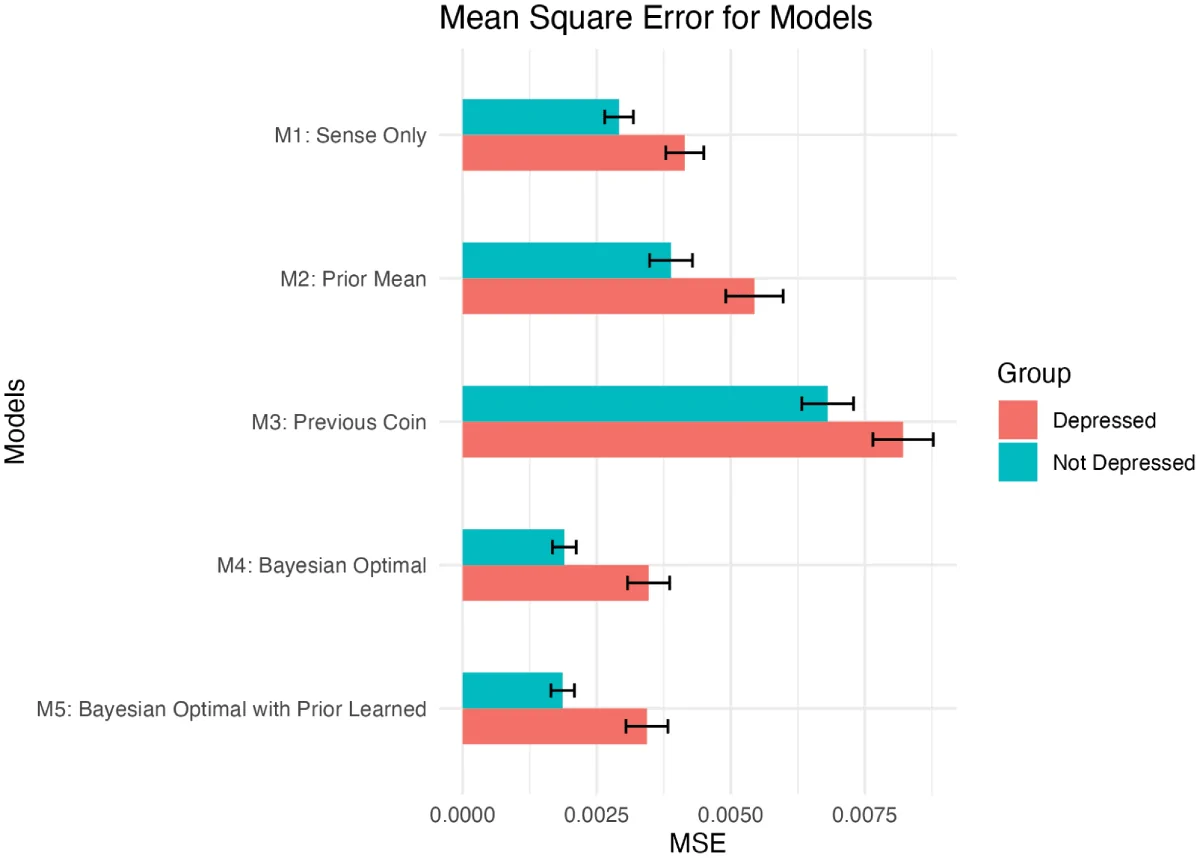
Model performance per group. The bars represent the mean squared errors (MSE), separated by groups. The better-performing models have smaller MSEs. Bayesian models (M4, M5) and the Sense Only model (M1) fit the non-depressed group significantly better than the depressed group (adjusted ps < 0.05). M2 and M3 did not differ significantly between groups.

Several factors beyond decision strategy could contribute to poorer model fit in the depressed group, including greater response variability, attentional lapses, or fatigue. Because depressed participants were more likely to complete the longer task version (600 trials), we tested whether the group difference in MSE could be explained by differential task exposure. When restricting the analysis to the subset of participants who completed the shorter version only (*n* = 7 depressed, *n* = 14 non-depressed), the pattern held: both Bayesian models and the Sense Only model continued to show significantly higher MSEs in the depressed group (all *p*s < 0.02), suggesting that the group difference in model fit is not driven by task length. Additionally, when including task version as a covariate in rank-transformed linear models across the full sample, the group effect remained significant for both Bayesian models (M4: *F*(1, 36) = 5.13, *p* = .030; M5: *F*(1, 36) = 5.46, *p* = .025), with version non-significant across all models (all *p*s > .59). These results confirm that the poorer model fit in the depressed group was not attributable to differences in task version. Nonetheless, the poorer fit may partly reflect heterogeneity within the depressed group or non-strategic sources of response noise rather than a qualitative difference in the decision-making approach.

## Discussion

We investigated whether depression alters the integration of prior knowledge and sensory (likelihood) information during sensorimotor decision-making under uncertainty, by independently manipulating uncertainty in both information sources. At a broad level, overall sensory reliance was comparable across groups. Both depressed and non-depressed participants downweighted noisier sensory cues and performed best when prior and sensory information were both reliable. Closer inspection, however, revealed some possible group differences. Although Bayesian models fit behavior better than heuristic alternatives in both groups, overall model fit was poorer in the depressed group (higher MSEs), suggesting that depression may be associated with additional noise and/or slightly attenuates precision-weighting rather than abolishing Bayesian-like integration. While both groups decreased reliance on prior information as it became less reliable, non-depressed participants showed a larger adjustment in sensory weighting than depressed participants as prior uncertainty changed, although this difference was not statistically significant. Together, these findings suggest that depression does not prevent the use of broadly Bayesian decision strategies, but may reduce sensitivity to changes in prior reliability.

Our findings speak to a related question: whether depression fundamentally disrupts Bayesian-like decision-making. Consistent with our findings, growing computational work suggests that depression does not eliminate Bayesian-like inference but alters the balance between priors and new evidence. Across a broad task battery, Mukherjee et al., 2020 reported that reward- and punishment-learning measures classified major depressive disorder status with 72% accuracy, consistent with deviations from normative integration across multiple domains (Mukherjee et al., 2020). Dimensional evidence in non-clinical samples similarly suggests that higher depressive symptoms predict less normative inference across perceptual and value-based tasks (Benwell et al., 2022), and that elevated decision uncertainty in approach–avoidance conflict can increase the discrepancy between observed choices and Bayesian-like predictions (Smith et al., 2023). A systematic review of computational modeling studies further concluded that depressive disorders are characterized by rigid priors and blunted updating across stable and volatile environments (Gibbs-Dean et al., 2023). Within this broader context, our model comparisons showed that Bayes-optimal accounts outperformed heuristic alternatives for both groups, yet Bayesian models left larger residual errors in the depressed group. This pattern is consistent with the possibility that depression weakens adaptive precision-weighting, making behavior harder to capture with normative Bayesian-like observers without introducing additional mechanisms (e.g., increased response noise, altered decision policies, or state-dependent shifts in precision). In other words, depressed individuals may remain qualitatively Bayesian-like, while showing reduced flexibility when the reliability structure of the environment changes. In practical terms, this suggests that the basic process of combining what we expect with what we observe still works in depression. Everyday judgments (for example, how much to trust a first impression of someone against what we already know about them, or whether to change our mind after an ambiguous outcome) require weighing prior belief against current evidence, and depressed participants in our study did this in a way that normative models described well. When depression is described in terms of distorted or rigid beliefs, the problem may lie in the beliefs, not in how they are weighed against new evidence. What differs may be the type of the prior, or how readily its reliability is updated, rather than the integration process. This distinction matters: if the weighing process operates normally but takes more pessimistic expectations as its input, then the expectations themselves may be the more useful target for intervention.

Within this broadly preserved pattern, we also observed a more subtle difference in adaptive flexibility. Although both groups relied more on priors as they became reliable, depressed participants showed attenuated shifts in sensory weighting as prior uncertainty changed. Our trial-level analyses capture this possible suboptimal adjustment: when the prior became unreliable, non-depressed participants increased sensory weighting by approximately *Δ* = 0.173, whereas depressed participants increased sensory weighting by only *Δ* = 0.088, showing an almost twofold difference in the magnitude of reweighting; although this group difference was only marginally significant (*p* = 0.051) and should be interpreted with caution given the modest sample. This potential reduced flexibility aligns with recent computational accounts of belief formation and updating in depression. Large-scale modeling work suggests that higher depression scores are associated with more negative priors (i.e., more pessimistic expectations about outcomes), whereas the basic rate of updating may be relatively preserved (Gagne et al., 2022). Other studies indicate that depression is associated with more negative updating relative to normative Bayesian expectations, whereas non-depressed individuals show an optimism bias, weighting good news more strongly than bad (Feldmann et al., 2023; Korn et al., 2014). A complementary perspective proposes that the critical impairment may be a broader reduction in integrating novel evidence once priors are established, yielding rigid expectations that are less responsive to incoming data (Kube, 2023; Kube et al., 2019).

Our results also revealed an unexpected response-time pattern. A substantial literature links depression to slower responding on cognitive tasks, with meta-analytic evidence for robust reaction time slowing, particularly in melancholic subtypes characterized by psychomotor retardation (Pulcu & Browning, 2019; Sobin & Sackeim, 1997). Latent-process modeling of perceptual decision-making further suggests that depressed individuals may set wider decision boundaries and accumulate evidence more slowly (Lawlor et al., 2020; Pitliya et al., 2022). Despite this, neither group showed slower responding overall. Instead, both groups responded faster when prior uncertainty was large. One framework that may help explain this pattern is the idea that uncertainty modulates response policy. Paulus and Yu (2012) proposed that uncertainty can promote action selection that terminates uncertainty-related distress, even at the cost of precision. Related evidence suggests that individuals high in Intolerance of Uncertainty may prefer immediate, less valuable options that reduce waiting in uncertainty, although effects have been mixed across studies (Cobos et al., 2024; Luhmann et al., 2011). Applied to our paradigm, elevated prior uncertainty may have been experienced as aversive by both depressed and non-depressed participants, promoting earlier response commitment to curtail the uncertain state rather than prolong deliberation. Alternatively, when prior information is unreliable, participants may simply have less information to deliberate over, reducing the computational demands of integrating two sources and thereby speeding responses. This interpretation is compatible with evidence that uncertainty-related distress can shape response policy independent of general processing speed (Aylward et al., 2019). In this view, psychomotor slowing and uncertainty-driven response acceleration are conceptually separable: the former reflects global cognitive-motor efficiency, whereas the latter reflects a shift in response policy driven by the informational context. Put differently, changes in response policy under uncertainty could yield faster overt responses in specific contexts without contradicting broader evidence for depressive slowing.

Several limitations affect our conclusions. First, the pilot sample size was modest, which likely limited statistical power to detect smaller group differences and constrained generalizability. As a pilot study, we did not conduct an a priori power analysis, and future confirmatory work should use power-based sampling to test the observed effect sizes. Second, grouping was based on BDI-II scores rather than referral status or formal diagnosis, such that some participants recruited as controls met the depressed criterion. This dimensional approach captures the continuum of depressive symptoms, but also yields a heterogeneous “depressed” group in terms of treatment-seeking status and clinical history, limiting direct comparability with studies using diagnostically defined samples. Third, the task placed relatively low demands on executive control. Cognitive deficits in depression are more reliably observed on tasks with higher executive demands (Snyder, 2013), and the relatively simple visuomotor judgments required here may not have sufficiently taxed the control processes where depression-related impairments are most pronounced. Relatedly, discrepancies with prior work on processing speed may reflect differences in task demands and cognitive domain (Elderkin-Thompson et al., 2007; Snyder, 2013), as well as heterogeneity in depression subtypes, symptom profiles, and comorbidities (Douglas & Porter, 2009; Millan et al., 2012). We also did not test the impact of medication status, depression subtype, or education, which may have obscured more subtle effects and should be addressed in larger samples.

Altogether, these results indicate that individuals with depression can engage in sensorimotor decision-making that is broadly consistent with Bayesian-like principles. Both depressed and non-depressed participants combined sensory cues with learned priors, giving more weight to sensory information when sensory information was more reliable and/or when the prior was more uncertain. The key difference appears to be quantitative rather than qualitative: when prior reliability changed, behavior in both groups was better captured by Bayesian models than by heuristic alternatives, though model fit was somewhat poorer in the depressed group. Thus, depressed individuals can learn and use priors and sensory evidence in a broadly Bayesian-like manner, and any reduction the flexibility of precision-weighting when the reliability of prior information changes appears to be a matter of degree rather than kind.

## Additional File

The additional file for this article can be found as follows:

10.5334/cpsy.195.s1 Supplemental Information. Supplemental Information including Supplemental Methods and Supplemental Results, as well as associated Supplemental Figures.
