# Supplementary material for: Investigating Sensorimotor Decision-Making in Depressed Individuals Using a Bayesian Approach
Source: Comput Psychiatr. 2026 Oct 9;10(1):202–18. doi: 10.5334/cpsy.195 (PMC13651772; doi:10.5334/cpsy.195)
Supplement: Supplementary file 1 [file cpsy-10-1-195-s1.pdf]

Supplemental Information for

Investigating Sensorimotor Decision-Making in Depressed Individuals: A Bayesian Approach

University of Minnesota, Twin Cities

Supplementary Methods

Participants (cont.)

Participants were classified as depressed or non-depressed based on BDI-II total score, with scores >13 indicating depression and scores ≤13 indicating no depression. This cutoff followed standard BDI-II severity bands (0–13 minimal, 14–19 mild, 20–28 moderate, 29–63 severe). In addition, a meta-analysis reported optimal cutoffs of 18.18 for psychiatric samples and 12.9 for healthy and somatic samples (von Glischinski et al., 2019). Because our sample combined referred patients and community volunteers, we applied a single cutoff of 13 across groups. Using a cutoff of 14.5 (also recommended as a general optimal cutoff in the same meta-analysis) produced an identical group split in our data.

Behavioral Analysis Through Computational Modeling

Bayes' theorem offers a structured methodology for combining prior knowledge and current sensory (likelihood) data, thereby optimizing the reduction of uncertainty. If we assume that both the prior and likelihood distributions are normal, then the posterior estimate can be mathematically represented as:

$$X_{posterior\ estimate} = {\sigma_L}^2/({\sigma_L}^2 + {\sigma_P}^2)\mu_P + {\sigma_P}^2/({\sigma_L}^2 + {\sigma_P}^2)\mu_L \quad (1)$$

Within the context of our study, $X_{posterior\ estimate}$ signifies a participant's estimation of the coin's location. The variance (${\sigma_L}^2$) and mean (μ_L) of the likelihood, or sensory input, are derived from the dot-cloud, while the variance (${\sigma_P}^2$) of the prior emerges from the coin position distribution. The experiment designated the screen's center (0.5 in screen coordinates) as the average prior position, so μ_P was always 0.5 in this experiment. The likelihood's mean, represented by the dot cluster's centroid, is dynamic across different trials.

When regressing the participant's estimated coin position on the central point of the displayed cloud of dots for each trial, we obtain a slope termed the 'sensory weight.' This represents the extent to which participants rely on likelihood or sensory information. If the sensory weight aligns with the Bayes optimal model, as previously detailed, it suggests participants are estimating in a manner consistent with the Bayesian optimal formula:

$$SW = {\sigma_P}^2/({\sigma_L}^2 + {\sigma_P}^2) \quad (2)$$

A slope approaching 1 indicates a predominant reliance on sensory information, suggesting participants primarily use the dot cloud to estimate the coin's location. Conversely, a slope close to 0 indicates limited dependence on sensory information, indicating that participants are not relying on sensory data when estimating the coin's location. The sensory weight offers insights into the strategies participants employ in the task and the balance between their reliance on sensory data and prior information. Importantly, by design, "Bayesian" sensory weights should fall between 0 and 1. Any sensory weight substantially deviating from the Bayesian-predicted value ${\sigma_P}^2/({\sigma_L}^2 + {\sigma_P}^2)$ hints that the participant's behavior does not align with Bayesian predictions.

Bayesian-optimal Sensory Weight Calculation

Assuming the imposed variances, the statistically optimal sensory weight as informed by Bayesian theory can be deduced from the formula $SW_{optimal} = {\sigma_P}^2/({\sigma_L}^2 + {\sigma_P}^2)$. Under the

assumptions of this model, the participants' perceived variances for the prior and likelihood are considered equivalent to the experimentally-imposed variances. Specifically, the prior variance was set at either $\sigma_P{}^2 = 0.025^2$ for small prior uncertainty conditions or $\sigma_P{}^2 = 0.075^2$ for large prior uncertainty conditions. The sensory (or likelihood) variance, $\sigma_L{}^2$, was either small ($\sigma_l{}^2 = 0.025^2{*}5$) or large variance ($\sigma_L{}^2 = 0.075^2 * 5$). It is important to emphasize that this is a simplification; the actual perceived variances by participants might be different than the given values. However, since each participant's subjective variance remains unobservable, these experimentally imposed variances serve as a valuable benchmark to evaluate real-world behavior.

In our study, sensory weights were calculated for each participant across four distinct experimental conditions: “pl” (small prior uncertainty, small likelihood uncertainty), “pL” (small prior uncertainty, large likelihood uncertainty), “Pl” (large prior uncertainty, small likelihood uncertainty), and “PL” (large prior uncertainty, large likelihood uncertainty). These sensory weights were then compared against the Bayesian-optimal sensory weights, determined based on the experimentally imposed prior and likelihood variances. Specifically, the theoretical Bayesian-optimal sensory weights for conditions pl, pL, Pl, and PL were 0.5, 0.1, 0.9, and 0.5, respectively, as derived from the equation provided earlier. We computed the mean squared error (MSE) between their observed sensory weights and the corresponding Bayesian-optimal values across the four conditions. This was calculated as the average of the squared differences: ((observed_pl – 0.5)^2 + (observed_pL – 0.1)^2 + (observed_Pl – 0.9)^2 + (observed_PL – 0.5)^2) ÷ 4.

Trial-by-trial Sensory Weight Calculations

Equation (1) and (2) above can be rearranged to derive a trial-by-trial sensory weight:

trial est P L P

$$X_{est} = (1 - SW_{trial})\mu_P + SW_{trial}\mu_L$$
$$X_{est} = \mu_P - SW_{trial}\mu_P + SW_{trial}\mu_L$$
$$X_{est} - \mu_P = -SW_{trial}\mu_P + SW_{trial}\mu_L$$
$$X_{est} - \mu_P = SW_{trial}(\mu_L - \mu_P)$$
$$SW_{trial} = (X_{est} - \mu_P)/(\mu_L - \mu_P)$$

Where X_{est} is the participant's estimated coin position on that trial (i.e., where they placed the net), μ_p is the mean of the prior (assumed to always be 0.5, corresponding to the centre of the screen), and μ_L is the mean of the likelihood (calculated here as the centroid of the dot cloud on that trial). When the prior and likelihood means are very close to one another, SW_{trial} can become arbitrarily large; we therefore applied a logistic transformation $SW_{trial}(1/(1 + e^{-SWtrial}))$ to bound the trial-by-trial weight between 0 and 1.

Computational Modeling: Fitted Models

We provided a brief description of the model fitting rationale in the results section, and here we listed the model details of all the models we fitted to the data, including model assumptions and calculations.

<u>Model 1 (sense-only model)</u>: This model assumes the sensory weight is 1, meaning the participant's coin location estimate is equal to that trial's dot cloud centroid.

Model 2 (prior mean-only model): Here, the sensory weight is set to 0, with the weight on prior information being 1. Consequently, participants consistently estimate the coin's location based on the established prior mean, μ_P, which is 0.5.

Model 3 (previous coin location model): For the first trial, this model uses the prior mean (0.5) for the coin's estimated location and uses the previous coin position in all subsequent trials. It assumes that participants use a different type of prior information (previous coin location) to estimate the coin's location.

Model 4 (Bayes-optimal with task parameters): To determine where the participant would likely position the net during each trial (representing their optimal prediction of the coin's location for that specific trial), this model employs equation (1) combined with experimentally defined prior variance values. Specifically, $\sigma_P{}^2 = 0.025^2$ is used in blocks with small prior uncertainty, and $\sigma_P{}^2 = 0.075^2$ is used in blocks with large prior uncertainty.

Model 5 (Bayes-optimal with prior learned per trial): This model assumes that prior variance is learned trial to trial, and the participant is constantly updating their own prior variance instead of using the experimentally imposed one shown in Model 4.

$$\sum_{t}^{t-1} (coinPosition_{t-1} - \mu_P)/(t-1)$$

μ_P is the experimental mean of the prior (0.5), t is the current trial number, and $coinPosition_{t-1}$is the coin position at the last trial; the sum is cumulative, so the estimate on each trial incorporates all previously observed coin positions rather than only the most recent one. Deviations are taken from the fixed prior mean of 0.5 rather than from a running sample mean, as only the variance of the prior was manipulated. For the first trial, given that participants haven't seen any coin location yet, the prior variance is set as 0.5^2, which corresponds to the squared distance from the screen centre to the edge of the response range and therefore represents a maximally uncertain prior over possible coin positions. The variance estimate accumulates continuously across the entire task and is not reset when the prior variance changes between blocks, to avoid assuming that participants know/remember that prior variance may change between blocks, and to take into account learning effects from the previous block.

To assess the effectiveness of each estimation model, we computed the Mean Squared Error (MSE) and the coefficient of determination (R^2) values for all five models. MSE offers a clear picture of the average squared differences between the model's predicted coin location and the actual coin location chosen by the participants, while R^2 providing a measure of how well the predicted coin locations by the model align with the actual estimated coin location. Note that the MSEs and R^2 were calculated on the full data to get an accurate evaluation of the model's performance. A smaller MSE value indicated a better-fitting model, and a higher R^2value would indicate that the model is a better fit for the data. See Table 1 in the main manuscript for a summary of model performance. We follow the modeling approach of Trapp and Vilares (2020), where these models are described in further detail.

Supplementary Results

Sensitivity to Likelihood and Prior Uncertainty by Groups

To assess participants' sensitivity to changes in prior and likelihood uncertainties, we first calculated each participant's sensory weight in all four experimental conditions. Sensitivity to prior was determined by averaging the difference in sensory weights between the two conditions of prior uncertainty, computed $((SW_{Pl} + SW_{PL}) - (SW_{pl} + SW_{pL}))/2$; sensitivity to likelihood uncertainty was defined as the average difference in sensory weights between the two conditions of likelihood uncertainty, given by $((SW_{pl} + SW_{Pl}) - (SW_{pL} + SW_{PL}))/2$. Independent-sample t-tests were subsequently conducted to test for differences between the depressed and non-depressed groups.

Sensitivity to likelihood uncertainty (non-depressed mean: 0.19; depressed mean: 0.14) and to prior uncertainty (non-depressed mean: 0.17; depressed mean: 0.09) were all significantly greater than zero (one-sample t-tests, $p < 0.05$). When comparing the sensitivities between depressed and non-depressed individuals, the mean sensitivity to prior uncertainty was lower in the depressed group compared to the non-depressed group, suggesting that the depressed group may react less to changes in prior uncertainty, but this difference was not statistically significant ($t(37) = -2.0$, $p = 0.051$; Figure S1, left). For likelihood uncertainty, the depressed group also showed slightly lower sensitivity compared to the non-depressed group, but again, the results did not reach statistical significance ($t(37) = -1.80$, $p = 0.08$; Figure S1, right).

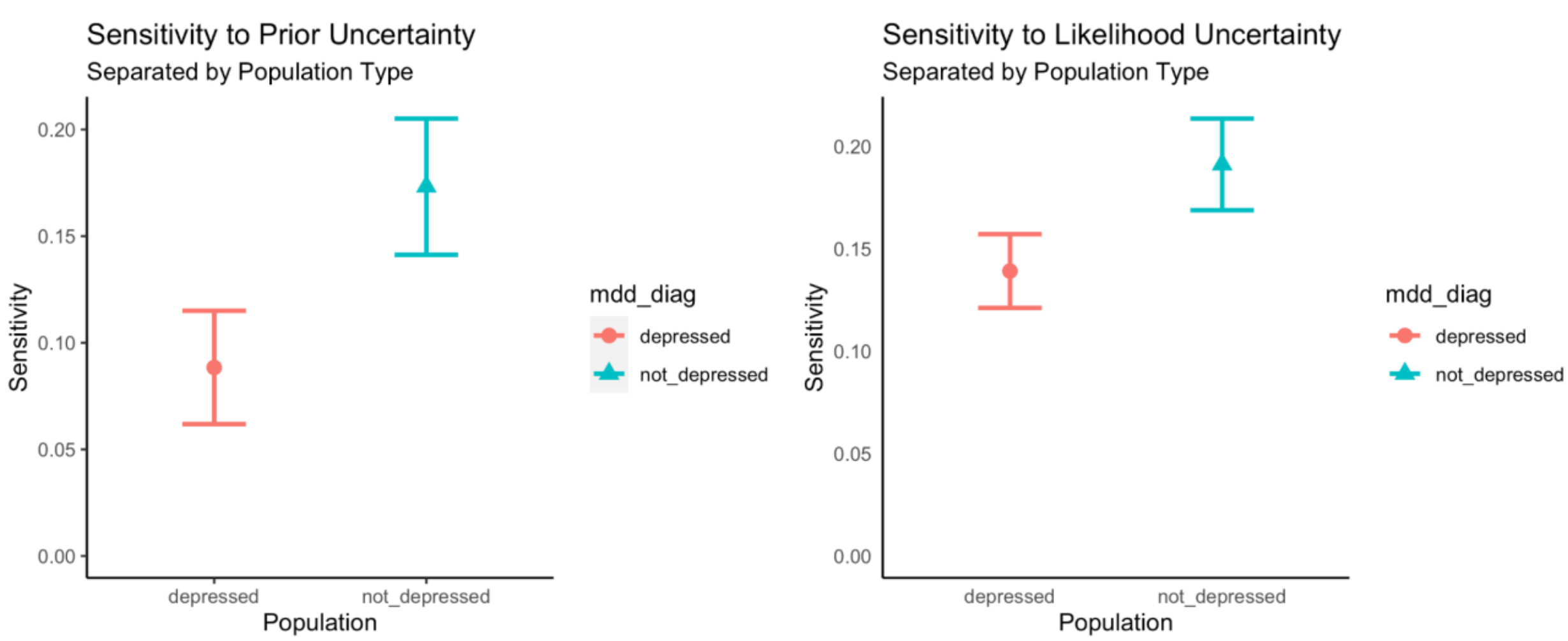

Figure S1. Sensitivity to likelihood and prior uncertainty comparison for depressed and non-depressed groups. Left: Sensitivity to prior uncertainty ± standard error of the mean (s.e.m.) separated by groups, calculated as the average difference in sensory weights between the low prior uncertainty conditions (reliable prior information) and the high prior uncertainty conditions (unreliable prior information). Depressed participants displayed slightly reduced sensitivity to prior uncertainty compared to non-depressed participants; however, this difference was not significant. Right: Sensitivity to likelihood uncertainty ± standard error of the mean (s.e.m.), separated by groups, type. This is calculated as the average difference in sensory weights between the low sensory uncertainty conditions (reliable sensory information) and the high sensory uncertainty conditions (unreliable sensory information). Depressed participants demonstrated slightly reduced, but not statistically significant, sensitivity to sensory uncertainty compared to non-depressed participants.

Comparing Participants' Sensory Weights with Bayesian-optimal Sensory Weights

While both groups qualitatively tracked Bayesian expectations and the non-depressed group showed numerically smaller deviations from Bayesian optimals (mean MSE = 0.066) compared to the depressed group (mean MSE = 0.105), the difference was not significant (Welch's $t(31.6) = 1.73$, $p = 0.093$). Looking per condition, a series of one-sample t-tests was conducted to assess whether participants' sensory weights significantly deviated from these optimal values. For the non-depressed group, significant deviations were observed in the pL condition ($t(14) = 5.64$, $p < .001$) and the Pl condition ($t(14) = -3.05$, $p = .009$), indicating that non-depressed participants' sensory weights differed from the Bayesian predictions, specifically when either the prior or likelihood uncertainty was large. In contrast, no significant deviation was found for the pl ($t(14) = 1.51$, $p = .154$) or PL ($t(14) = 1.47$, $p = .165$) conditions. For the depressed group, sensory weights significantly deviated from Bayesian-optimal values in three conditions: pl ($t(23) = 2.36$, $p = .027$), pL ($t(23) = 8.28$, $p < .001$), and Pl ($t(23) = -4.00$, $p < .001$). No significant deviation was observed for the PL condition ($t(23) = 1.77$, $p = .091$). Similar to the non-depressed group, these findings suggest that depressed participants showed departures from Bayesian-optimal sensory weights, particularly when one source of information became unreliable. Despite these quantitative deviations, the broader behavioral pattern across both groups remained qualitatively consistent with Bayesian predictions. Specifically, participants adapted their sensory weighting in response to changes in uncertainty, avoiding over-reliance on either prior or sensory information when the other became unreliable. This suggests that, regardless of group status, participants displayed adaptive behavior qualitatively (but not quantitatively) aligned with Bayesian principles (see Figure 5 in the main manuscript).

Block-wise Adaptation Rate by Prior Variance

We also explored the speed at which individuals altered their sensory weight under varying prior uncertainty conditions and examined whether depression affected their block-wise updating of sensory weighting, defined as the change in sensory weight over time, while adapting strategies to different prior uncertainties. As prior variance alternates between blocks, shifts in sensory weight over time should reflect changes in participants' perceived prior variance. To investigate this, trials were divided into different prior uncertainty blocks (e.g., participants were assigned to either four blocks of small-large-small-large, or large-small-large-small prior uncertainty blocks; Figure S2) with 10 time bins within each block. The average sensory weight was calculated for each time bin across 12-15 trials, based on the total number of trials completed by participants. Block-wise adaptation rates of sensory weights (or change in sensory weight over time) were computed within each block, and the adaptation rates between the depressed and non-depressed groups were compared to determine potential differences in the rate at which they learned to adjust their sensory weight to the prevailing prior uncertainty condition. As shown in Figure S2, both the depressed and non-depressed groups initially demonstrated a capacity to rely less on sensory information in the small prior uncertainty block (Figure S2, top panel) and more on sensory information in the large prior uncertainty block (Figure S2, bottom panel), as evidenced by a gradual shift in sensory weight across time bins within the block. No significant differences were observed between the adaptation rates of the depressed and non-depressed groups (Top panel 1st block: $t(22) = 0.00$, Bonferroni-adjusted $p = 0.99$; Bottom panel 1st block: $t(13) = 0.52$, adjusted $p = 0.61$). Similarly, for blocks 2-4, no significant differences were identified in the block-wise adaptation rate of sensory weighting

(slope of change in sensory weight) between the control and depressed groups (all adjusted $p > 0.05$). These findings suggest that both depressed and non-depressed participants rapidly adapted their strategies when initially undertaking the task, and that both groups remained comparably responsive to prior volatility over time.

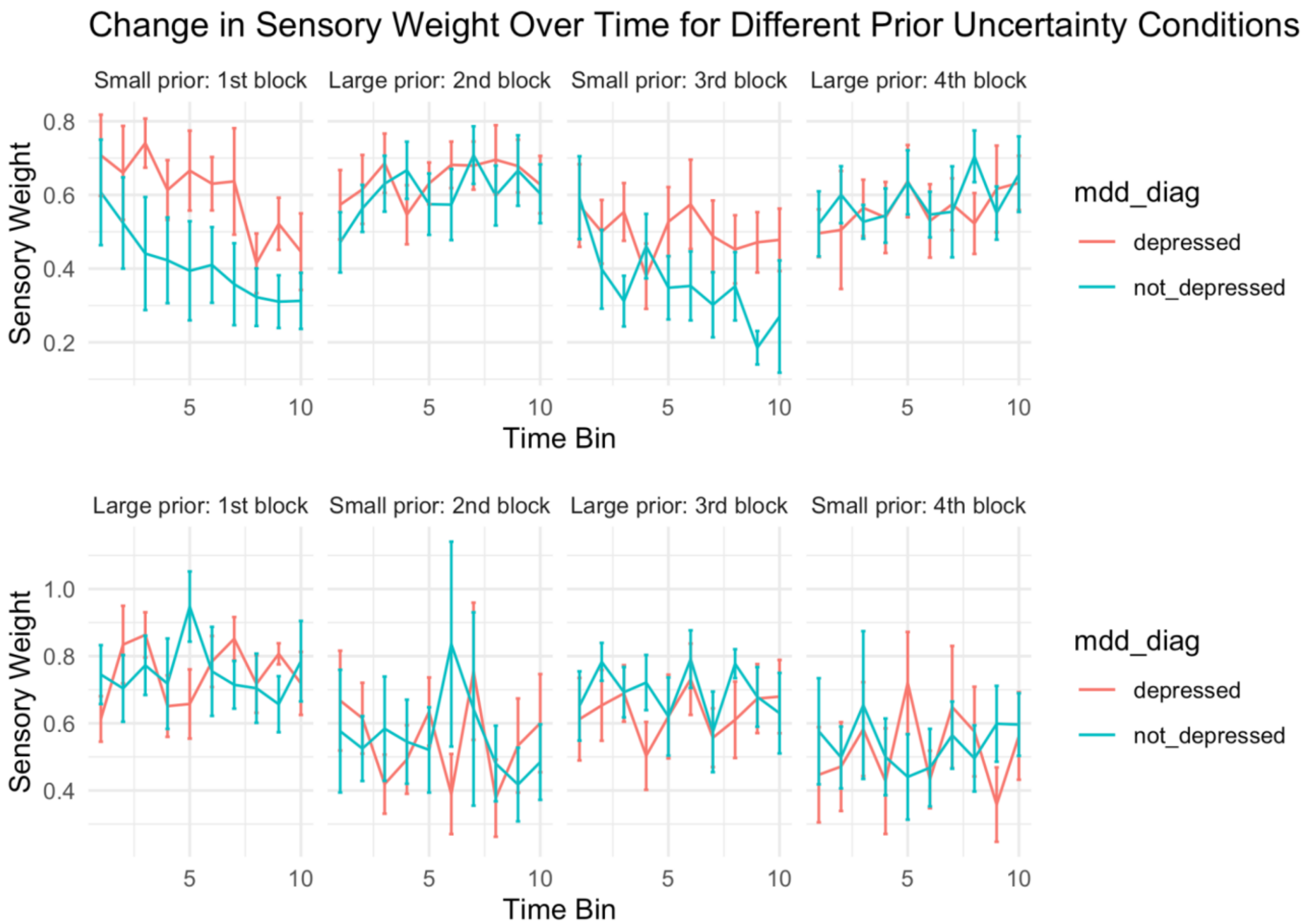

Figure S2. Block-wise adaptation rate, or change in sensory weight across time (± s.e.m.) as individuals adapted to different prior uncertainties. Participants were assigned to different prior uncertainty blocks, with sensory weight slopes calculated within each block. Both depressed and non-depressed participants demonstrated a capacity to adjust sensory weight based on prior uncertainty (e.g., Top panel 1st block). No significant differences were observed between the adaptation rates of the depressed and non-depressed groups, suggesting that depressed participants showed comparable block-wise adaptation rates of sensory weights to non-depressed participants.

Reaction to trial-by-trial changes in likelihood uncertainty

We next asked whether participants adjusted their reliance on sensory evidence on a trial-by-trial basis as likelihood uncertainty changed (see the Supplemental Methods for details of the trial-by-trial sensory weight calculation). Trial-level adaptation was quantified as the Pearson correlation between sensory uncertainty on a given trial and the relative weight placed on sensory information on that trial, averaged within participant; if participants adjusted their

weighting in the direction predicted by Bayesian integration, we would expect a negative correlation between sensory uncertainty and participants' sensory weight placed on that trial. Both groups showed a negative mean correlation (small in magnitude) that differed significantly from zero (non-depressed: $M = -0.14$, $t(14) = -7.61$, $p < 0.001$; depressed: $M = -0.11$, $t(23) = -8.33$, $p < 0.001$), indicating that participants in both groups relied less on sensory information as it became less reliable (Figure S3). An independent-samples t-test showed no group difference ($t(37) = 1.32$, $p = 0.20$). Although these correlations were small in magnitude, they were consistent in sign and reliably different from zero, indicating that both groups engaged in rapid reweighting in the direction predicted by the normative model. The modest size of these correlations likely reflects the noise inherent in single-trial estimation and does not necessarily indicate weak adaptation; block-level analyses confirmed robust sensitivity to likelihood uncertainty in both groups (see Results in the main text). Together, these results indicate that both depressed and non-depressed participants adjusted their strategy rapidly in response to trial-by-trial changes in likelihood uncertainty in a manner broadly consistent with Bayesian integration.

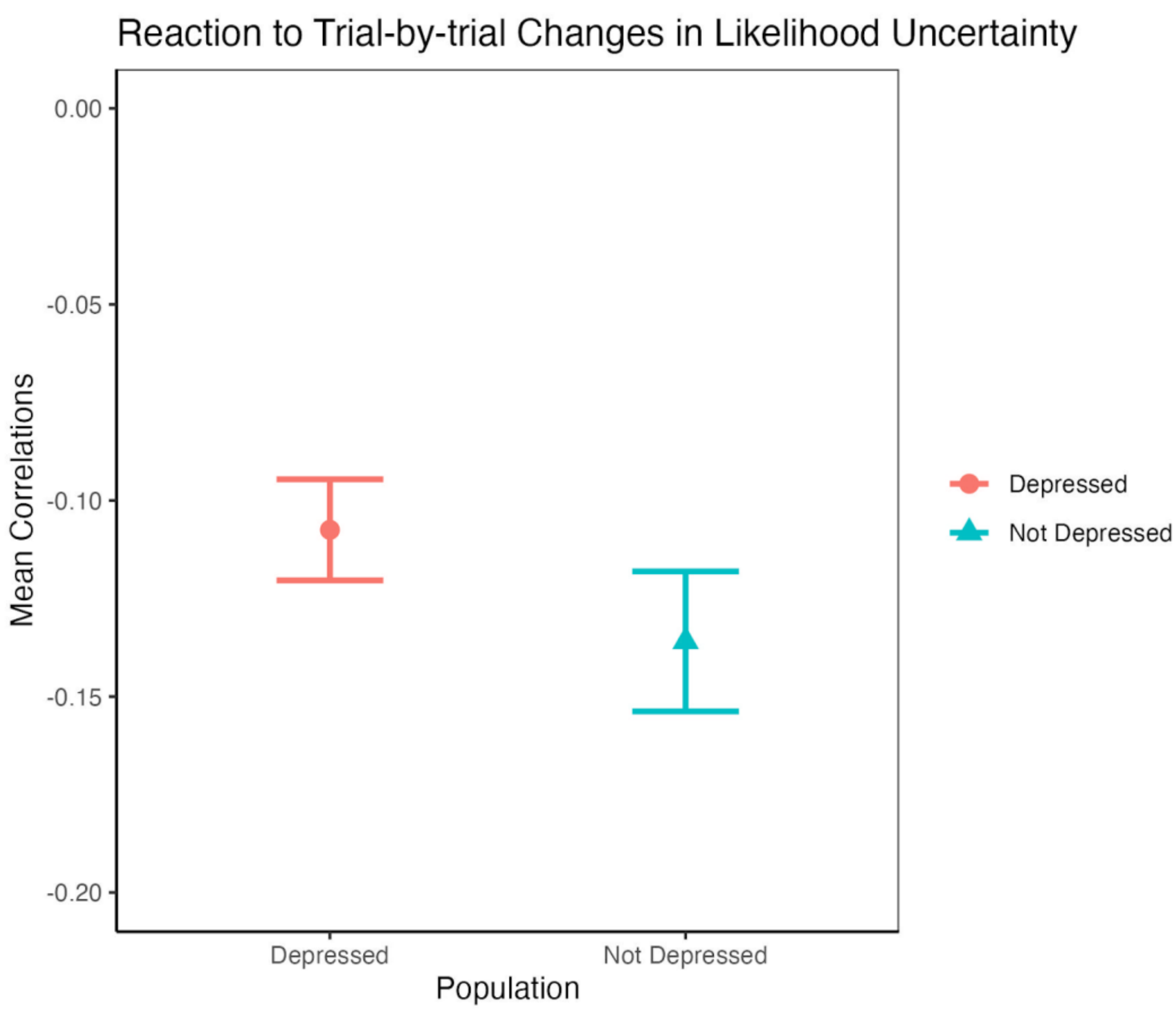

Figure S3. Trial-by-trial adaptation to likelihood uncertainty (mean Pearson correlation ± s.e.m.). Correlations reflect the association between a trial's sensory uncertainty (s.d. of the dot cloud) and the relative weight placed on sensory information on that trial. Both groups showed negative correlations, with no significant difference between group means.

Correlation between Sensory Reliance and Depression Severity

We explored whether depression severity influenced participants' sensory reliance in the coin-catching task. To do this, we conducted a series of correlation tests to examine the relationships between participants' depression scores on BDI-II and their sensory weights, prior sensitivity, and likelihood sensitivity. We excluded two depressed participants from this analysis due to missing data. As non-depressed participants may still have some degree of depression or depressive experiences (although not clinically depressed), instead of categorizing participants into depressed and non-depressed groups and running separate correlation tests, in this analysis, we combined participants from both groups (n = 39). A Spearman's correlation revealed a very weak, non-significant correlation ($r(37) = 0.038$, $p = 0.82$) between the BDI-II total score and sensory weight. In addition, we found negative, non-significant correlations between BDI-II scores and participants' sensitivity to likelihood uncertainty ($r(37) = -0.21$, $p = 0.20$) and sensitivity to prior uncertainty ($r(37) = -0.22$, $p = 0.18$).

Response Time: Age, Gender, BDI-II scores, and Task Versions as Covariates

In assessing the impact of groups (depressed vs. non-depressed), prior, and likelihood on response time, a linear mixed-effects analysis was performed with age as a covariate. Response times were log-transformed prior to analysis to reduce positive skew. The main effects revealed a significant influence of prior on response time ($F_{1, 20835} = 40.69$, $p < 0.001$), but no significant effect of group ($F_{1, 36} = 0.41$, $p = 0.525$), likelihood ($F_{1, 20835} = 2.17$, $p = 0.140$), or age ($F_{1, 36} = 2.68$, $p = 0.110$). The group × prior interaction remained marginal ($F_{1, 20835} = 3.36$, $p = 0.067$), consistent with the pattern observed in the primary analysis. The inclusion of age as a covariate did not substantively change any results.

Similarly, we used gender as a covariate in the model with prior, likelihood, and group as fixed factors. Our results showed that gender had no significant effect on response time ($F_{2, 35} = 1.79$, $p = 0.182$). All other results were consistent with the primary analysis.

When BDI-II severity rating was included as a covariate, the results remained unchanged: there was no significant effect of severity ($F_{1, 36} = 1.98$, $p = 0.168$), and the main effect of prior uncertainty and the marginal prior × group interaction were preserved ($p < 0.001$ and $p = 0.067$, respectively). Similarly, when BDI-II total score was used as a covariate, there was no significant effect of total score ($F_{1, 36} = 3.20$, $p = 0.082$), and the pattern of results was again consistent with the primary analysis.

When task version was included as a covariate, the results remained largely unchanged: there was no significant effect of version ($F_{1, 36} = 1.56$, $p = 0.220$), and the main effect of prior uncertainty and the marginal prior × group interaction both remained consistent ($p < 0.001$ and $p = 0.067$, respectively).

References

Trapp, S., & Vilares, I. (2020). Bayesian decision-making under stress-preserved weighting of prior and likelihood information. *Scientific Reports*, *10*(1), 21456.
