# Supplementary material for: Investigating Sensorimotor Decision-Making in Depressed Individuals Using a Bayesian Approach
Source: Comput Psychiatr. 2026 Oct 9;10(1):202–18. doi: 10.5334/cpsy.195 (PMC13651772; doi:10.5334/cpsy.195)
Supplement: Peer review history

# Reviewer report: Round 1, Reviewer D

**DOI:** 10.5334/cpsy.195.r1

## Recommendation:

Accept with Minor Revision

## Comments/Explanation (required). Please include references to any minor or major revisions required, as well as addressing any issues with language clarity. Authors will see these comments:

I found this manuscript highly interesting . Investigating depression from a Bayesian observer perspective offers a compelling way to examine whether alterations associated with depression extend beyond affective and reward-related decision-making to more fundamental sensorimotor inference - this is an important step! In particular, the independent manipulation of uncertainty in prior and sensory information provides an informative framework for studying how flexibly individuals adjust the relative weighting of different information sources. I therefore found the general research question original, theoretically relevant, and well suited to Computational Psychiatry.

The manuscript is clearly written overall, and the authors have made a considerable effort to explain and unpack the modeling approach in the Supplemental Information. I also found the Discussion thoughtful and appropriately balanced, particularly in acknowledging that poorer model fit in the depressed group could reflect several mechanisms, including altered precision-weighting, greater response variability, attentional lapses, or fatigue. More generally, the Discussion provides a useful and nuanced contextualization of the findings within current accounts of belief updating and cognitive flexibility in depression.

My overall recommendation would be minor revision. However, I should emphasize that computational modeling is not my primary area of expertise, and I do not feel fully equipped to evaluate every statistical and modeling decision in sufficient technical depth. Given how central these analyses are to the manuscript’s conclusions, I would find it helpful if the editor also obtained input from a reviewer with more specialized expertise in computational modeling. My comments below therefore primarily reflect questions that remained open to me as an interested but non-specialist reader. Clarifying these points may also help make the manuscript more accessible to the broader readership of Computational Psychiatry.

1. Could the authors clarify what conclusions can be drawn from the comparison between Models M1–M3 and the Bayesian Models M4 and M5? M1–M3 appear to represent relatively extreme strategies in which participants rely exclusively on sensory information, the prior mean, or the previous coin location. In contrast, M4 and M5 combine both prior and sensory information. Does the superior performance of M4 and M5 therefore demonstrate specifically Bayesian or uncertainty-sensitive integration, or primarily that participants combine more than one source of information? Would it be useful to compare the Bayesian models with a more competitive alternative that also combines prior and sensory information, but uses a fixed weighting that does not vary with uncertainty? Alternatively, could the authors clarify or qualify the interpretation of the current model comparison?

2. Could the authors guide the reader more explicitly through the derivation of the trial-by-trial sensory weight? In particular, I found the transition from Equation 1 to the trial-level equation in the Supplemental Information difficult to reconstruct. A more step-by-step derivation would be helpful, including an explanation of the rationale for the subsequent transformation, how it affects the resulting values, and how the transformed quantity should be interpreted as a sensory weight.

3. Could the authors clarify the relationship between the participant-level sensitivity analysis in the Supplemental Information and the mixed-effects interaction reported in the main manuscript? The Supplemental Information states that sensitivity to prior uncertainty was lower in the depressed group, but that this difference was not statistically significant, (t(37)=-2.0, p=.051). In contrast, the main manuscript reports a significant prior uncertainty × group interaction, (F(1,111)=7.00, p=.009). As these findings appear to address a closely related group difference, it would be helpful to explain more explicitly how the tested quantities and statistical models differ and why they lead to different inferential conclusions.

4. Could the authors provide the complete random-effects structure of the mixed-effects model? In particular, were participant-specific random slopes included for prior and likelihood uncertainty? If the model included only a random intercept, could the authors comment on whether the central prior uncertainty × group interaction remains robust when individual differences in the within-participant effect of prior uncertainty are represented in the random-effects structure?

5. Could the authors clarify which analysis should be considered the primary statistical test of the conclusion that depressed participants show reduced sensitivity to changes in prior reliability? This seems to be the central empirical finding of the manuscript, and a short explanation of why the mixed-effects interaction is preferred over the participant-level sensitivity comparison would help readers evaluate the evidence.

6. Could some elements of the model descriptions in the Supplemental Information be made more explicit? For example, a clearer explanation of how the prior variance is updated in Model M5, how the initial value was chosen, and whether the learning process is reset when the thrower changes between blocks would improve reproducibility and accessibility.

7. The Discussion already provides a strong theoretical contextualization. Could the authors additionally offer a somewhat more psychologically tangible interpretation of the central effect? For example, what might reduced sensitivity to changes in prior reliability mean outside this specific sensorimotor task? I regard this as a suggestion rather than a substantive weakness, but it could help readers appreciate the broader psychological relevance of the findings.

Overall, I found this to be a thoughtful and promising study that applies a valuable computational perspective to an understudied aspect of depression. The authors have already made a substantial effort to explain the analyses and to acknowledge alternative interpretations. Clarifying the scope of the model comparison, guiding the reader more explicitly through the trial-level derivation, and explaining the relationship between the two statistical tests would further strengthen the manuscript.

## Consent to Publish

Consent Given

# Reviewer report: Round 1, Reviewer E

**DOI:** 10.5334/cpsy.195.r2

## Recommendation:

Accept (No Revisions Necessary)

## Comments/Explanation (required). Please include references to any minor or major revisions required, as well as addressing any issues with language clarity. Authors will see these comments:

This manuscript presents a clear and thoughtful investigation of how depression may affect the integration of prior and sensory information during sensorimotor decision-making. The study is well suited to the scope of Computational Psychiatry, and the task provides an interesting way of examining Bayesian-like decision processes outside affective or reward-based contexts. I particularly appreciated the care taken in examining possible influences on the results. The authors considered task version, recruitment stream, group composition, age, and gender, and repeated relevant analyses using alternative specifications and restricted samples. The central finding remained consistent across these checks. The manuscript is also appropriately transparent about the limitations of the pilot sample and the parameter-free models, as well as alternative explanations for the poorer model fit in the depressed group. Overall, the paper is clearly written, the conclusions are appropriately measured, and the analyses support the main interpretation. I have no substantive requests for revision and recommend acceptance in its current form.

## Consent to Publish

Consent Given

# Reviewer report: Round 1, Author Response

**DOI:** 10.5334/cpsy.195.r3

## Editorial Revision Requests:

Dear Prof. Dr. Jamalabadi,

We thank the editor and reviewers for the thoughtful comments, which allowed us to improve our paper. Following the suggested comments, we now: 1) Give more details on the fitted models, clarify the model comparison and offer suggestions for other models, 2) Give more detail and include a step-by-step derivation of the trial-level sensory weight derivation in the supplemental information and 3) Fit a mixed-effects model with both random intercepts and random slopes, and highlight the differences and similarities between the results from the different mixed-effects and the participant-level sensitivity comparison. Furthermore, we updated some parts of the results and discussion to accommodate this extra analysis as well as to include a more psychologically tangible interpretation of our main effects.

Below, we reply to each reviewer comment individually, with the reviewer’s comments in *italics*, and our answer below. We believe that our manuscript has improved following the reviewers’ comments and subsequent changes, and we hope you find it suitable for publication at *Computational Psychiatry*.

Kind regards,

Iris Vilares (on behalf of all authors)

**Reviewer 1**

***Reviewer 1***:

*Recommendation: Revisions Required*

***Recommendation***:

*Accept with Minor Revision*

## Reviewer D:

***Comments/Explanation (required). Please include references to any minor or major revisions required, as well as addressing any issues with language clarity. Authors will see these comments***:

*I found this manuscript highly interesting. Investigating depression from a Bayesian observer perspective offers a compelling way to examine whether alterations associated with depression extend beyond affective and reward-related decision-making to more fundamental sensorimotor inference - this is an important step! In particular, the independent manipulation of uncertainty in prior and sensory information provides an informative framework for studying how flexibly individuals adjust the relative weighting of different information sources. I therefore found the general research question original, theoretically relevant, and well suited to Computational Psychiatry*.

*The manuscript is clearly written overall, and the authors have made a considerable effort to explain and unpack the modeling approach in the Supplemental Information. I also found the Discussion thoughtful and appropriately balanced, particularly in acknowledging that poorer model fit in the depressed group could reflect several mechanisms, including altered precision-weighting, greater response variability, attentional lapses, or fatigue. More generally, the Discussion provides a useful and nuanced contextualization of the findings within current accounts of belief updating and cognitive flexibility in depression*.

## Author’s response

Response: We thank the reviewer for the positive comments, as well as for the additional suggestions to improve the manuscript.

## Reviewer D:

*My overall recommendation would be minor revision. However, I should emphasize that computational modeling is not my primary area of expertise, and I do not feel fully equipped to evaluate every statistical and modeling decision in sufficient technical depth. Given how central these analyses are to the manuscript’s conclusions, I would find it helpful if the editor also obtained input from a reviewer with more specialized expertise in computational modeling. My comments below therefore primarily reflect questions that remained open to me as an interested but non-specialist reader. Clarifying these points may also help make the manuscript more accessible to the broader readership of Computational Psychiatry*.

*1. Could the authors clarify what conclusions can be drawn from the comparison between Models M1–M3 and the Bayesian Models M4 and M5? M1–M3 appear to represent relatively extreme strategies in which participants rely exclusively on sensory information, the prior mean, or the previous coin location. In contrast, M4 and M5 combine both prior and sensory information. Does the superior performance of M4 and M5 therefore demonstrate specifically Bayesian or uncertainty-sensitive integration, or primarily that participants combine more than one source of information? Would it be useful to compare the Bayesian models with a more competitive alternative that also combines prior and sensory information, but uses a fixed weighting that does not vary with uncertainty? Alternatively, could the authors clarify or qualify the interpretation of the current model comparison*?

## Author’s response

Response: We thank the reviewer for this thoughtful comment and for highlighting the need to clarify the interpretation of our computational modelling results. We agree that, because Models M1–M3 each rely exclusively on a single source of information, it is unsurprising that a model assigning non-zero weight to both the prior and the likelihood would outperform them. Consequently, our model comparison does not, by itself, demonstrate that participants are implementing Bayesian or uncertainty-sensitive integration, but rather provides evidence that participants combine information from more than one source.

Our intention was not to claim that the superiority of M4 and M5 uniquely establishes Bayesian computation. Instead, we sought to show that Bayesian models provide a substantially better account of participants’ behaviour than simple heuristic strategies that rely on a single information source. As the reviewer suggests, a model that combines prior and likelihood information using a fixed weighting, independent of uncertainty, would provide a more stringent comparison and would help distinguish Bayesian uncertainty-sensitive integration from simpler combination strategies.

To avoid overinterpreting our findings, we have revised the manuscript to make this distinction explicit. We now state that the superior performance of the Bayesian models demonstrates that participants integrate both prior and sensory information more effectively than is captured by the heuristic models considered here, “but it does not rule out other combination strategies that also use both sources (e.g., a fixed mixture that does not adjust to uncertainty)”. We have also identified direct comparison with fixed-weight integration models as an important direction for future work

## Reviewer D:

*2. Could the authors guide the reader more explicitly through the derivation of the trial-by-trial sensory weight? In particular, I found the transition from Equation 1 to the trial-level equation in the Supplemental Information difficult to reconstruct. A more step-by-step derivation would be helpful, including an explanation of the rationale for the subsequent transformation, how it affects the resulting values, and how the transformed quantity should be interpreted as a sensory weight*.

## Author’s response

Response: We thank the reviewer for catching this. Reviewing the Supplemental Information, we noticed that the numerator contained a sign error: the equation should read *SW* = (*X_est_ – μ_P_*)/(*μ_L_ – μ_P_*) rather than *SW_trial_* = (*X_est_ + μ_P_*)/(*μ_L_ – μ_P_*). We have corrected the equation and now include the full step-by-step derivation from Equation (1) in the Supplemental Information for clarity. We thank the reviewer again for their careful reading.

## Author’s response

*Comments 3-5*

Response: We thank the reviewer for these three closely related comments; we refit a model that includes random intercepts and by-participant random slopes for prior and likelihood uncertainty, and we address comments 3-5 below. We have also revised the Results section accordingly.

## Reviewer D:

*3. Could the authors clarify the relationship between the participant-level sensitivity analysis in the Supplemental Information and the mixed-effects interaction reported in the main manuscript? The Supplemental Information states that sensitivity to prior uncertainty was lower in the depressed group, but that this difference was not statistically significant, (t(37)=-2.0, p=.051). In contrast, the main manuscript reports a significant prior uncertainty × group interaction, (F(1,111)=7.00, p=.009). As these findings appear to address a closely related group difference, it would be helpful to explain more explicitly how the tested quantities and statistical models differ and why they lead to different inferential conclusions*.

## Author’s response

Response: The reviewer is correct that the two analyses assess the same underlying effect using different statistical approaches. The participant-level sensitivity to prior uncertainty reported in the Supplemental Information is the effect of prior uncertainty averaged across likelihood conditions, thus only one value per participant is used. In contrast, the mixed-effects model reported in the main text includes the 4 conditions per participant, and thus allows testing for different factors in a single model (group, prior, and likelihood), taking into account participant-level effects. The discrepancy the reviewer mentioned between the two tests is due to the different way residual errors and random-effects are specified (the mixed-effects model includes random participant effects, which the sensitivity analysis doesn’t), which can, in theory, lead to different results/p-values, as within-participant variability information is lost when data is aggregated (Brown, 2021). While a mixed-effects model is generally preferred when there are multiple factors being studied and repeated measures (as is our case), we also wanted to note what the effect would be when doing a simpler, straightforward t-test. Following the reviewer’s suggestion, we also refitted the mixed-effects model to include both random intercepts and random slopes. Under this revised specification, the two analyses now reach the same conclusion: the mixed-effects interaction is marginally significant (F(1,37) = 4.07, p = 0.051), matching the participant-level sensitivity t-test (t(37) = –2.0, p = 0.051). We have revised the results accordingly and added a sentence explicitly linking the two analyses (see also the answers to points 4 and 5 below).

## Reviewer D:

*4. Could the authors provide the complete random-effects structure of the mixed-effects model? In particular, were participant-specific random slopes included for prior and likelihood uncertainty? If the model included only a random intercept, could the authors comment on whether the central prior uncertainty × group interaction remains robust when individual differences in the within-participant effect of prior uncertainty are represented in the random-effects structure*?

## Author’s response

Response: We thank the reviewer for this important comment and for highlighting the need to clarify the random-effects structure of our mixed-effects models. The original model included by-participant random intercepts only; both prior and likelihood uncertainty were manipulated within participants, assuming that all participants share the same within-participant effects. Following the reviewer’s suggestion, we refitted the model to include by-participant random slopes for prior and likelihood uncertainty. This model provided a significantly better fit (χ²(5) = 25.45, p < 0.001; AIC = –177.9 vs. –162.5), indicating substantial individual differences in how participants adjusted their weighting in response to changes in uncertainty that were not captured by the original specification.

Under the random-slopes model, the pattern of fixed effects remained unchanged. However, the prior uncertainty × group interaction was attenuated and became marginally significant (F(1,37) = 4.07, p = 0.051) compared with the original model (F(1,111) = 7.00, p = 0.009). The revised denominator degrees of freedom reflect the number of participants contributing independent within-participant contrasts. We have updated the Results section to include the random-slopes model, as we agree that it provides the most appropriate and conservative representation of the data.

## Reviewer D:

*5. Could the authors clarify which analysis should be considered the primary statistical test of the conclusion that depressed participants show reduced sensitivity to changes in prior reliability? This seems to be the central empirical finding of the manuscript, and a short explanation of why the mixed-effects interaction is preferred over the participant-level sensitivity comparison would help readers evaluate the evidence*.

## Author’s response

Response: We thank the reviewer for this helpful suggestion. The primary statistical analysis is the mixed-effects model because it reflects the factorial repeated-measures design, estimating the effects of prior uncertainty, likelihood uncertainty, and group simultaneously while accounting for within-participant dependence. The participant-level sensitivity analysis provides a useful summary measure of the same effect, but it collapses across likelihood conditions and does not fully utilise the experimental design.

It is worth noting that the central conclusion of the manuscript is that individuals with depression continue to integrate prior and sensory information in a broadly Bayesian-like manner, and this conclusion is unaffected by the revised analysis. With respect to the group difference in sensitivity to prior uncertainty, following the revised random-effects specification, the two analyses now lead to the same conclusion. We report the mixed-effects model as the primary analysis and note in the Results that it is consistent with the participant-level analysis presented in the Supplemental Information.

We have also revised the Abstract and Discussion to reflect the marginal significance of this group difference. Importantly, the estimated effect size is unchanged in both magnitude and direction (Δ = 0.085, 95% CI [–0.0004, 0.170]); non-depressed participants adjusted their sensory weights by approximately twice as much as depressed participants (Δ = 0.173 vs. 0.088), and both groups individually showed significant sensitivity to prior uncertainty (both p < 0.01). We now describe the between- group difference as marginal and have noted in the Limitations that the modest sample size limited power to detect an effect of this magnitude.

## Reviewer D:

*6. Could some elements of the model descriptions in the Supplemental Information be made more explicit? For example, a clearer explanation of how the prior variance is updated in Model M5, how the initial value was chosen, and whether the learning process is reset when the thrower changes between blocks would improve reproducibility and accessibility*.

## Author’s response

Response: We thank the reviewer for this helpful suggestion. We have revised the description of Model 5 in the Supplemental Information to make the implementation explicit, including how the prior variance is updated, how the initial value is defined, and whether learning is reset between blocks.

Specifically, the prior variance on each trial is calculated as the mean squared deviation of *all* previously revealed coin positions from the fixed prior mean of 0.5. The estimate is cumulative rather than recursive on the previous trial, and deviations are computed relative to the imposed prior mean rather than a running sample mean, because only the prior variance was manipulated experimentally and participants were explicitly told about the prior mean (informed that “*the person that is throwing the coin is trying to throw to the middle of the screen*”). The initial prior variance was set to 0.5², and it was chosen because 0.5 is the distance from the screen center to the edge of the response range, so this represents a maximally uncertain prior before any coin locations had been observed. Finally, the estimate accumulates continuously throughout the experiment and is not reset at block transitions.

## Reviewer D:

7. The Discussion already provides a strong theoretical contextualization. Could the authors additionally offer a somewhat more psychologically tangible interpretation of the central effect? For example, what might reduced sensitivity to changes in prior reliability mean outside this specific sensorimotor task? I regard this as a suggestion rather than a substantive weakness, but it could help readers appreciate the broader psychological relevance of the findings.

## Author’s response

Response: We thank the reviewer for this helpful suggestion. We have expanded the Discussion to provide a more psychologically concrete interpretation of our findings.

Specifically, we now note that the basic process of integrating prior expectations with incoming sensory evidence appears to be largely preserved in depression, and that this computational process underlies many everyday judgements. We illustrate this with examples such as deciding how much to revise a first impression of someone in light of new information, or whether to update an expectation following an ambiguous outcome. We also suggest that, if depression is characterised by rigid or negatively biased beliefs, the critical difficulty may lie less in the ability to combine prior beliefs with new evidence per se than in the nature of those beliefs, or in how flexibly their reliability is updated. Finally, we briefly discuss the potential clinical relevance of this distinction, while emphasising that these interpretations are necessarily tentative given the scope of the present study.

## Reviewer D:

*Overall, I found this to be a thoughtful and promising study that applies a valuable computational perspective to an understudied aspect of depression. The authors have already made a substantial effort to explain the analyses and to acknowledge alternative interpretations. Clarifying the scope of the model comparison, guiding the reader more explicitly through the trial-level derivation, and explaining the relationship between the two statistical tests would further strengthen the manuscript*.

## Author’s response

Response: We thank the reviewer for the useful comments and suggestions on the manuscript, which we incorporated. We believe that our paper is now strengthened after the reviewer’s comments, and we thank you for your time and dedication to this process.
